# Supplementary material for: Impact of COVID-19 on mental health of primary healthcare workers in Pakistan: lessons from a qualitative inquiry
Source: BMJ Open. 2022 Dec 8;12(12):e065941. doi: 10.1136/bmjopen-2022-065941 (PMC9742844; doi:10.1136/bmjopen-2022-065941)
Supplement: Author's manuscript [file bmjopen-2022-065941.draft_revisions.pdf]

BMJ Open is committed to open peer review. As part of this commitment we make the peer review history of every article we publish publicly available.

When an article is published we post the peer reviewers' comments and the authors' responses online. We also post the versions of the paper that were used during peer review. These are the versions that the peer review comments apply to.

The versions of the paper that follow are the versions that were submitted during the peer review process. They are not the versions of record or the final published versions. They should not be cited or distributed as the published version of this manuscript.

BMJ Open is an open access journal and the full, final, typeset and author-corrected version of record of the manuscript is available on our site with no access controls, subscription charges or pay-per-view fees (http://bmjopen.bmj.com).

If you have any questions on BMJ Open's open peer review process please email info.bmjopen@bmj.com

BMJ Open

Psychosocial and mental health consequences of COVID19 among primary healthcare workers in Pakistan: lessons from a qualitative inquiry

Journal:	*BMJ Open*
Manuscript ID	bmjopen-2022-065941
Article Type:	Original research
Date Submitted by the Author:	25-Jun-2022
Complete List of Authors:	Hameed, Waqas; The Aga Khan University, Community Health Sciences<br>Avan, B; London School of Hygiene and Tropical Medicine,<br>Feroz, Anam Shahil; Aga Khan University, Department of Community Health Sciences<br>Khan, Bushra; University of Karachi, Department of Psychology<br>Fatmi, Zafar; Aga Khan University, Department of Community Health Sciences<br>Jafri, Hussain; Fatima Jinnah Medical University<br>Wassan, Mansoor Ali; Government of Sindh<br>Siddiqi, Sameen; Aga Khan University Medical College Pakistan, Community Health Sciences Department
Keywords:	MENTAL HEALTH, HEALTH SERVICES ADMINISTRATION & MANAGEMENT, COVID-19

SCHOLARONE™
Manuscripts

I, the Submitting Author has the right to grant and does grant on behalf of all authors of the Work (as defined in the below author licence), an exclusive licence and/or a non-exclusive licence for contributions from authors who are: i) UK Crown employees; ii) where BMJ has agreed a CC-BY licence shall apply, and/or iii) in accordance with the terms applicable for US Federal Government officers or employees acting as part of their official duties; on a worldwide, perpetual, irrevocable, royalty-free basis to BMJ Publishing Group Ltd ("BMJ") its licensees and where the relevant Journal is co-owned by BMJ to the co-owners of the Journal, to publish the Work in this journal and any other BMJ products and to exploit all rights, as set out in our licence.

The Submitting Author accepts and understands that any supply made under these terms is made by BMJ to the Submitting Author unless you are acting as an employee on behalf of your employer or a postgraduate student of an affiliated institution which is paying any applicable article publishing charge ("APC") for Open Access articles. Where the Submitting Author wishes to make the Work available on an Open Access basis (and intends to pay the relevant APC), the terms of reuse of such Open Access shall be governed by a Creative Commons licence – details of these licences and which Creative Commons licence will apply to this Work are set out in our licence referred to above.

Other than as permitted in any relevant BMJ Author's Self Archiving Policies, I confirm this Work has not been accepted for publication elsewhere, is not being considered for publication elsewhere and does not duplicate material already published. I confirm all authors consent to publication of this Work and authorise the granting of this licence.

For peer review only

Bismillah hir Rahman nir Raheem

Title:
Psychosocial and mental health consequences of COVID19 among primary healthcare workers in Pakistan: lessons from a qualitative inquiry

Short title: COVID19 and mental health of primary healthcare workers

Authors:
Waqas Hameed[1], Bilal Iqbal Avan[2], Anam Shahil Feroz[1], Bushra Khan[3], Zafar Fatmi[1], Hussain Jafri[4], Mansoor Ali Wassan[5], Sameen Siddiqi[1]

[1]Department of Community Health Sciences, Aga Khan University, Karachi, Pakistan
[2]Department of Clinical Research, London School of Hygiene and Tropical Medicine, London, United Kingdom
[3]Department of Psychology, University of Karachi, Sindh, Pakistan
[4]Fatima Jinnah Medical University, Lahore, Pakistan
[5]Department of Health, Government of Sindh, Pakistan

Correspondence:
Bilal Iqbal Avan
Associate Professor
Department of Clinical Research
London School of Hygiene and Tropical Medicine
Keppel St, London WC1E 7HT, United Kingdom
bilal.avan@lshtm.ac.uk

For peer review only

For peer review only - http://bmjopen.bmj.com/site/about/guidelines.xhtml

Abstract

Objectives

The existing literature regarding the mental health consequences of COVID19 among healthcare workers resolves predominately around specialised hospital settings, while neglecting primary healthcare workers who are the first point of contact for patients. In view of negligible evidence, this study explored the psychological impact of COVID19, health system response, and sought suggestions and recommendations from the primary healthcare workers (PHCWs) to address their psychological needs in pandemic crisis.

Methods

Employing a qualitative exploratory design, we telephonically conducted 47 in-depth interviews with PHCWs in 42 health facilities across 15 districts in Sindh and Punjab provinces of Pakistan. A combination of inductive and deductive approach was used for data analyses.

Results

There was immense fear, stress and anxiety among PHCWs of being infected and infecting their families at the beginning of this outbreak and its peak which tapered off over time. It was triggered by lack of information about the virus and its management, false rumours, media-hype, lack of personal protective measures (PPEs), non-cooperation from patients and community people. Awareness raising trainings and PPEs provided by the healthcare system, while social support from co-workers and supervisors was instrumental in addressing their psychological needs. Additionally, they recommended appreciation and recognition, and provision of psychosocial support from mental health professionals.

Conclusion

Primary healthcare system should be prepared to provide timely informational (e.g. continuous updates in training and guidelines), instrumental (e.g. provision of personal protective equipment, appreciation and recognition), organisational (e.g. safe and conducive work environment), and emotional and psychological support (e.g. frequent or needs-based session from mental health professionals) to PHCWs in order to mitigate the psychological impact of pandemic crisis.

Keywords: COVID19, primary healthcare, mental health, psychological, health system

For peer review only - http://bmjopen.bmj.com/site/about/guidelines.xhtml

Introduction:

The novel coronavirus disease 2019 (COVID-19) has swept across the world.[1] As of September 28, 2021, the virus has affected nearly 232 million people and accounted for approximately 4.75 million deaths worldwide[2], including 115,000 healthcare workers (HCWs)[3]. This large-scale unprecedented public health catastrophe, placed the frontline HCWs, who are directly involved with all aspects of care of COVID-19 patients, at risk of developing psychological distress and other mental health symptoms.[4] Studies conducted thus far provide evidence of the adverse impact of COVID19 on mental health and well-being of HCWs, primarily those who are involved in providing specialised care to COVID19 patients[5]. According to a systematic review, the pooled prevalence of depression, anxiety and post-traumatic stress disorder among HCWs across 21 (low to high income) countries hovered around 22%[5]. The prevalence of such mental health disorders vary according to sex, age, and role of HCWs[6,7].

While these studies made substantial contribution to inform strategies in alleviating the burden of psychological distress among HCWs, the prime focus has been on professionals who are working in hospital settings and are primarily engaged in the provision of specialised care to COVID19 patients[6,7]. In contrast, primary healthcare workers (PHCWs) have been managing a major share of the burden of COVID19 related care[8] by being the first point of contact for a broad spectrum of COVID-19 patients, whether suspected, confirmed (with or without symptoms), or exposed to case(s), as well as those with non-COVID19 health needs[9]. Studies indicate that some 80% of cases are mild and the majority of moderate cases seek PHC services as the entry point for getting medical care[10].

Community health workers (CHWs) also played a distinct role during COVID19, in lower middle-income countries (LMICs), by being involved in community awareness, engagement and sensitisation and contact tracing though at the cost of routine service delivery [11]. These newly assigned tasks during pandemic not only exposed them to increased risk of infection, but they also experienced stigmatisation, isolation or social ostracisation[11]. Ironically, they reportedly received inadequate support from health system in the form of personal protective equipment[12], and logistics and supportive supervision. Besides that, PHCWs are also responsible to manage the aftermath of contingencies and bearing the collateral damage caused by reallocating resources to COVID-19 whilst inadvertently hindering access to a wide range of primary care services[13,14]. This put them under stressful situations during this pandemic[15].

Despite the ongoing debate on how strengthening PHC (including CHWs) can help health systems adapt during the COVID-19 pandemic,[16,17] the significance and instrumental role of PHCWs in the times of COVID19 pandemic has received little attention of the global research community. A large majority of the research conducted on mental health consequences of COVID19 among HCWs has focused in the hospital settings[18]. Furthermore, there are few qualitative studies conducted that provide in-depth psychosocial experiences of HCWs, in general, as well as those working in PHC settings [19]. Even a recent effort to consolidate potential interventions to address mental health issues of HCWs during such outbreak is largely based evidence generated from HCWs working in specialised settings, while neglecting the psychological needs of and solutions for PHCWs [20].

Keeping in view of the role that PHCWs as alluded to above, we hypothesised that they may have suffered from similar mental health issues as faced by HCWs working in specialised healthcare settings. Furthermore, as COVID19 is expected to become endemic[21,22], the role of PHC becomes pivotal for its community-based information structure to identify risk groups, monitor compliance, provide preventive and promotional services, and contact tracing in this and similar future pandemic. Hence, their psychological needs, readiness and response become central to tackling

For peer review only - http://bmjopen.bmj.com/site/about/guidelines.xhtml

such issues. In order to better support PHCWs with their adapted role during COVID19, interventions are needed that could effectively cater to their mental health and psychosocial needs in the long run [23]. With this rationale, we set following research questions for this research that focuses on public-sector PHCWs in Pakistan.

Research questions:
1. What is the perceived psychological impact of COVID19 on PHCWs in Pakistan?
2. How health system has responded to the psychological needs of PHCWs?
3. What are the recommendations to support mental and psychosocial needs health of PHCWs in pandemic crisis?

Country Context:
Pakistan has a population of over 225 million inhabitants[24]. The first COVID19 case was identified in February 2020, and since then a total number of confirmed cases exceed 1.2 million along with 27,638 deaths has been registered (till September 28, 2021)[25]. The country has experienced four waves – each with different variant including delta, more recently[25]. With a focus on vulnerable groups of HCWs and elderly (65+ years) people, the country embarked on a nation-wide vaccine campaign for COVID19. The vaccine is now available for everyone except for children under the age of 12 years[25]. While several vaccines are safe and effective, there is strong emphasis on practicing preventive measures by the government.

Public-sector healthcare in Pakistan is delivered through a three-tiered health system. The primary health care provides services through a community-based component comprising over 100,000 CHWs and health facility component that includes almost 10,000 basic health units (BHUs), rural health centres (RHCs), maternal and child centres (MCH), and civil dispensaries[26]. The government of Pakistan has taken several measures to combat this deadly pneumonia virus[27] but it leaves a lot to be desired[28]. The health system of Pakistan is weak[29] and with critical shortage of health workforce, and is not prepared to sustain a major surge in COVID-19 cases[30]. Few studies have been conducted to assess the impact of COVID19 on mental health of HCWs; While these studies do identify symptoms of mental disorders, all have focused on tertiary level of health system [31-33].

Methods:
Study design and setting
A qualitative exploratory design was adopted using in-depth interview technique. Exploratory design enabled us to gather detailed description of HCWs experiences during the COVID19 pandemic. The study was conducted in 42 health facilities across 15 districts of Sindh and Punjab provinces of Pakistan. Districts where the cumulative number of COVID19 cases were high since the onset of this outbreak till date of survey were selected. Participants were selected using a criterion purposive sampling approach from randomly selected public health facilities stratified by districts and type of health facilities (BHUs and RHCs). We also collected contact details of affiliated community health workers (referred to as "Lady Health Workers"[34] hereafter) from the in-charge of the selected health facility.

Recruitment of study participants:
The study participants were classified into three groups: lady health workers (LHWs), facility-based health service providers (doctor, nurse, midwife, lady health visitor), and administrators/managers (medical superintendent, lady health supervisors). Even though administrators at the PHC also often directly involved in service provision, we kept this distinction to see any diversity in their responses as opposed to their counterparts. The study participants

For peer review only - http://bmjopen.bmj.com/site/about/guidelines.xhtml

were recruited after obtaining formal permission from the higher government authorities, by contacting administrators of the randomly selected health facility.

Interview guide:
A standard semi-structured guide was used to interview all study participants, which was developed following extensive review of literature and consultation. It comprised of five major themes: a) Impact of COVID19 on mental health and well-being of health care professionals; b) Mental health and psychosocial needs of health care professional in COVID19 crisis; c) Mechanisms available within healthcare system to address mental health and psychosocial needs; and d) Challenges encountered in addressing MH and psychosocial needs; and e) Suggestions and recommendations to address MH and psychosocial needs. Open-ended questions and probes encouraged free flow of information from participants to developed deeper understanding of their experiences. The interview guide was pre-tested and revised based on few interviews among comparable non-study[35,36].

Data collection:
The data collection took placed between August and October 2020. Owing to restricted mobility due to COVID19, a total of 47 IDIs (Sindh: 29; Punjab: 18) were conducted telephonically until data saturation was achieved[37]. In the first phone call, participant was informed about the study and scheduled an appropriate data and time for the interview, if the participant is busy. Subsequent call was made at the schedule time for the interview. Experienced and trained data collectors conducted the interviews in local language (Urdu) with background in sociology and psychology. All the calls were made from the Aga Khan University under close supervision of study investigators. Data analysis was concurrently performed to determine the point of saturation. On average, the interviews lasted for 25-35 minutes. With the consent of participants, all the interviews were audio-recorded on tablets using a built-in call recording feature in the tablets. Data collectors were also encouraged to maintain key interview notes. A linguistic expert later transcribed the recordings into English for analysis.

Data analysis:
All interview notes, recordings, and transcriptions were imported into the NVivo version 11.0 software for analyses[38]. We explored divergent and congruent views of three groups, that is, facility-based service providers, administrators, and lady health workers. Conventional content analysis technique was used to analyse data. Two independent researchers (WH and AF) applied inductive coding while reviewing the transcriptions in NVivo[38] and identified new emerging codes and categories apart from pre-identified themes mentioned in the interview guide. The discrepancies in the interpretation of codes and themes were discussed among the investigation team to reach a consensus. Independent review of data and coding allowed coherent interpretations that are grounded in the data[36]

Patient and public involvement
We sought inputs from target audience through pre-testing of interview guides to ensure comprehension of the questions. Furthermore, in consultation with provincial health departments we selected districts where COVID-19 cases were high. The focal person in each selected district was oriented about the study who connected our team with in-charge of health facility to avoid any miscommunication. Lastly, they also advised about the dissemination of research findings – that is, they specifically emphasis to share it through research briefs to reach wider audience in the country.

Ethical considerations

For peer review only - http://bmjopen.bmj.com/site/about/guidelines.xhtml

Due to the telephonic interview, verbal informed consent was taken from all study participants which included permission to audio-record their interviews and use anonymized quotes. All study participants were informed about the study purpose, their rights as study participants, confidentiality, and publication of study findings in anonymised manner. We shared official permission letters with study participants issued by respective health department of government of Punjab and Sindh provinces to build trust. Ethics approval was obtained from Aga Khan University (Ref. number: 2020-5186-11602) and National Bioethics Committee of Pakistan (Ref. Number: 4-87/COVID-45/NBC/20/393).

Consolidated criteria for reporting qualitative research (COREQ) guidelines were used to organise this paper[39].

Results:

A total of 47 IDIs were included in the analysis. Table 1 shows the socio-demographic characteristics of participants. The mean age was 43.2 (±10.9) years and had an experience of 14.4 (±9.4) years. Approximately half were female (44.7%), 55.3% were health managers, and about 51.1% were working at the Basic Health Unit.

Table 1: Characteristics of study participants

Characteristics	n (%)
**Sex**	
Female	21 (44.7)
Male	26 (55.3)
**Age in years**	
Mean (±SD)	43.2 (10.9)
**Designation**	
Health service providers (doctor, nurse/midwife)	16 (34.0)
Health managers (lady health supervisors, medical superintendent)	26 (55.3)
Lady Health Workers	5 (10.6)
**Type of health facility**	
Basic Health Unit	24 (51.1)
Rural Health Centre	23 (48.9)
**Total experience in years**	
Mean (±SD)	14.4 (9.4)

The main study findings are organised into three main themes: perceived psychological impact of COVID-19; support mechanisms to address their psychological needs; and suggestions recommendations made by HCWs. Where observed, we also presented contrasting views of service providers and health managers, highlighting the diversity in provincial perspective (table 2).

Table 2: Themes and Categories

Themes	Categories
Theme 1: Perceived psychological impact of Covid-19 on healthcare workers	1.1 Anxiety due to uncertainty of Covid-19 and media exaggeration<br>1.2 Fear of infection, isolation and stigma<br>1.3 Challenges regarding patients<br>1.4 Stress due to lack of availability of personal protective equipment<br>1.5 Stress due to excessive work-load

For peer review only - http://bmjopen.bmj.com/site/about/guidelines.xhtml

Theme 2: Available support mechanisms to address psychological needs of healthcare workers	2.1 Awareness raising of healthcare workers<br>2.2 Provision of personal protective equipment<br>2.3 Social support from co-workers and family members<br>2.4 Managerial support from supervisors
Theme 3: Mental health needs of healthcare workers and recommended solutions	3.1 Safe and conducive work environment<br>3.2 Awareness about evolving situation and corrective actions<br>3.3 Psychosocial support from mental health professionals<br>3.4 Appreciation and recognition of their work in the pandemic crisis

1. Psychological impact of COVID19 on healthcare workers

1.1 Anxiety due to uncertainty of Covid-19 and media exaggeration

All study participants reported experiences of immense fear and anxiety due to the uncertainties around COVID-19 at the onset of this outbreak in Pakistan. It was primarily triggered by lack of knowledge, prevalent misconceptions about COVID19, and being aware of its disastrous effect in the developed world. There was a perception that it was a fatal virus and whoever got it would die. Others sceptical about its existence like how long will this outbreak last and will they remain bound forever to the new operational changes in service delivery.

"At the beginning, the real fear among people was that - a rumour spread that, if God Forbids, this much dangerous disease, and someone gets it he will die". Health manager, Ghotki.

"There's a bit of worriedness, mental pressure, what to do what not to do, how to go, should we go to their house or not, these problems happens, mental stress occurs" Lady Health Worker, Jamshoro".

For many HCWs was not being able to distinguish between COVID-19 positive and negative people around them due to its asymptomatic characteristics and lack of testing facility was mentally disturbing. It made the HCWs reluctant to interact with patients and community members, and even with their contacts at the very beginning of the outbreak.

"We ourselves were also mentally disturbed, that how do we know, we are going on duties, on fields, we staffs are meeting each other so how do we know that who has corona symptoms and who does not". Lady Health Supervisor, Rawalpindi

1.2 Fear of infection, isolation and stigma

While most of the PHCWs were not directly involved in providing care to COVID19 patients, there was still some fear of being contracted with COVID19 during their interaction with patients at the beginning and during peak time. Similar fear existed among LHWs during their routine household and targeted visits for contact tracing.

"We were afraid that we might get infected or our families might get infected. So we spend all this time under stress. We were seeing that there were many positive cases in our neighbourhood and the news on media". Health Manager, Shaheed Benazirabad

For peer review only - http://bmjopen.bmj.com/site/about/guidelines.xhtml

The level of fear was considerably higher among a few HCWs who were seconded to the isolation centres, and those who had any chronic disease (e.g. diabetes) or had old parents or young children due to possible transmission.

“At that time, my female health workers were very disturbed mentally. She was like we have small kids how we will run out-patient services… how everything will be managed. Their families were also tensed that you are going on duties and you are putting us on danger as well”. Health Manager, Shaheed Benazirabad

These stressors and uncertainties of being infected also affected provider-patient interaction during the initial phase of the outbreak and as it peaked. A service provider shared that:

“When a person is mentally unwell and is feeling uncertain, then s/he wouldn’t even deliver what is normally expected of him/her. We were not able to take care of the patients then. We didn’t know whether the incoming patient is COVID positive or not. We just kept a distance and asked patient ailment. We had to save ourselves someway”. Service Provider, Faisalabad

Fear of isolation was not reported by many HCWs. Only a few HCWs reported to be stressed as they had to stay away from their family members, or by being alone.

“She [my co-worker] took her medicine regularly for 14 days at home. Then her [COVID19] test was conducted after 14 days and it came negative. But still she was very worried and tensed, and she was crying that she had to stay away from her kids”. Service Provider, Khairpur

Mixed responses were reported by the LHWs in terms of stigma faced by them. According to some LHWs, community members resisted their entry into the households; whereas, senior or experienced LHWs reported fewer such issues in the field.

During corona [peak] days, when we [I and my LHW] used to go out, people used to complain a lot. A huge pandemic has spread and you are coming to our house, so how do we know whether you have it [COVID19] or not; you can infect us. We felt this kind of resistance a lot in field even though we used to go with PPEs (sanitizers and masks)… at some places, sometimes it [people’s reaction] was normal and some people gave very positive responses. Lady Health Supervisor, Rawalpindi

1.3 Challenges regarding patients:

A major challenge that was a source of stress and frustration among HCWs was uncooperative patients. Facility-based staff were concerned that patients were reluctant to wear face-mask or to adhere physical distancing within the premises of health facilities, and at times, they became offensive when they were insisted to take protective measures for COVID19.

“Our stressor was our patients because they were not ready to understand the situation. They didn’t cooperate with us. They thought we didn’t want to treat them. We get the announcement done here in our local areas and also pasted the posters. We also wrote on the board outside to ‘wash your hands’ and ‘wear mask in hospital’; but we had to tackle a lot of troubles like these. Health Manager, Gujrat

Due to illiteracy, people did not believe in the existence of any viral outbreak, and they considered such protective measures as tactics of health workers to avoid service delivery.

For peer review only - http://bmjopen.bmj.com/site/about/guidelines.xhtml

"Only those women are worried who are a little bit educated but mostly aren't, 99% of people are don't have any fear and they don't believe in corona existence and they think that we are making them stupid". Health Manager, Mirpur Khas

Early on, another challenge faced by HCWs was to conduct COVID19 test of suspected patients. According to them, people were extremely reluctant to share any details or willing to undergo COVID19 test. This was primarily due to the prevalent myths and misconceptions regarding COVID19 at the beginning of this outbreak.

"People used to bring sticks to beat us because it is a rural area and villagers used to say that our patients didn't have anything and they asked us to go away. We had to call assistant commissioner of that area than he used to send police. After that we were able to take samples from them. It was mental torture for us. Believe me, when we tell people that we are about to take their samples, tell us your mobile number and Identification Card number etc. I swear they curse us and run away" Service Provider, Sargodha

1.4 Stress due to lack of availability of personal protective equipment

Lack of availability of personal protective equipment or supplies put HCWs under immense stress of being infected. Even a HCW who was seconded to a quarantine centre expressed concerns about unavailability of PPE.

"I had duty in quarantine for 3 months then I didn't have any kit or PPE, or mask or glove. I got a call and they told me to come on duty that day at 2 so I started crying because both my parents were home. We heard about doctors and XYZ deaths. I was scared and didn't have any protection kit". Service Provider, Sialkot

In the absence of PPEs, some of the HCWs made use of the scarf or linen to cover themselves and their patients' face; while majority of the HCWs reported to have it purchased on their own.

"Obviously, there weren't many masks available for us even and for patients. So any piece of cloth, or scarf, we use to make them cover their mouths and nose. Males sometimes used linen or face mask". Health Manager, Ghotki

1.5 Stress due to excessive work-load

Increased work-load was mainly the concern of field-based staff (LHWs and LHS) and for those who were given additional responsibilities like secondment to COVID19 isolation centre. Field teams had to locate suspected cases at a short notice. Due to lack of proper transportation, especially during lockdown, made things were quite stressful and exhaustive.

"Everybody was busy running to and fro because [COVID19] cases would come without notice. Doctor would get anxious and ask us to collect data and pictures [of patients]. We had to go to LHS and LHW. Lady Health Supervisor, Rawalpindi

On the contrary, the work load reduced considerably during the initial phase in the health facilities as people was reluctant to go to health facilities in the fear of COVID and certain prevalent myths.

"Yes, it (health facility) was working but patients were very less. Usually we have 200 or 300 patients but during corona we had only 30 or 40 patients. Even there was news circulating in news channels that poison injection were being given in the hospitals to corona patients. So people

For peer review only - http://bmjopen.bmj.com/site/about/guidelines.xhtml

were really afraid and due to these kinds of rumours they stopped coming to hospitals". Service Provider, Sargodha

2. Available support mechanism to address psychological needs of health workforce

2.1 Awareness raising of healthcare workers

All HCWs reported to have received awareness raising trainings that were organised by the department. The trainings primarily focused on preventive aspect of COVID19: characteristics of virus, signs and symptoms, modes of transmission, population at risk, preventive and protective measures, quarantine guidelines, and its testing.

"The purpose of training was to screen yourself, keep yourself safe, save the people of your organization, and also protect community; things that can control the disease. If a patient has symptoms, tell them where to do test and how it is done". Service Provider, Khairpur

Almost all the participants informed that they have not received any training on mental health except for one lady health supervisor.

"In trainings, we talked on all issues, right. But, the main issue that was not touched is people's mental condition during corona. Nobody ever tried to assess that amongst us. We have never ever tried to understand people mental level or their mental condition or our feelings". Lady Health Supervisor, Rawalpindi.

Over time, increased awareness and declining COVID19 cases mitigate the fear and stress among healthcare workers.

"Right now [health] workers are not depressed they are normal and doing their routine work. They were depressed and worried in the beginning but now they are normal due to awareness campaigns". Health manager, Shaheed Benazirabad

2.2 Provision of personal protective equipment

The PPEs were provided to the health workers with some delays, after few weeks of the outbreak in the country. The use of these PPEs, combined with other protective measures of COVID19, played a major role in reducing fear and stress among healthcare workers.

"We displayed the posters outside of hospitals - informing people not to come to the hospital without any reason. Maintain social distancing, wash hands, make soaps available everywhere and made hand sanitizers available. That's how we coped up with the stress. To be very frank, in the beginning everything was very difficult for me and for them [health workers]". Service Provider, Multan

"Firstly, protection, masks, shields etc. there was a shortage before but now they are available. There is also awareness now, we are seeing that there are fewer patients in Pakistan compared to other countries. All these things are effective for mental health". Service Provider, Sialkot

2.3 Social support from co-workers and family members

Provision of social support by co-workers - including colleagues and supervisors – emerged as a common response mechanism to mitigate the fear, stress and anxiety among HCWs. During the

For peer review only - http://bmjopen.bmj.com/site/about/guidelines.xhtml

early phase of this outbreak when stress, anxiety and fear was high, staff members used to discuss and provide social support to each other. Senior HCWs often used to enquire about challenges being faced by their colleagues, including if they are emotionally disturbed. They used to console them to help overcome any kind of fear, stress or anxiety pertaining to COVID19.

"We just provided them emotional support. I educated staff members that we are health care providers, how could we help people if all of us become a victim of psychological issues. We have to be strong because we are on frontline. We don't have to be fearful. Along with that we used to appreciate workers. What if they themselves become depressed then who would work. I told them that corona has no limit so we will have to live with it". Health Manager, Khairpur

A couple of HCWs acknowledged that they used to consult and take frequent advice from ex-physiatrist mates to overcome stress, and some received support from their family members.

"I was in contact with a psychiatrist so in that way I used to get constant help from there. I personally took many sessions". Service Provider, Faisalabad

"My husband kept on telling me that the night that is in the grave is not outside, so stop thinking about it and the major support that my husband gave me was it is a death by martyrdom so I used to get relaxed from that as well". Lady Health Supervisor, Rawalpindi

The emotional support was provided in several ways. Some of the consoling approaches were: a) reinforcement of the use of PPEs and COVID19 SOPs; b) reiterated the purpose of being a health worker; c) ensured frequent communication with the stressed person and giving assurance of being with them; d) motivated colleagues by giving their own example; e) encouraging others to keep themselves busy if they are stressed. Importantly, some used religious healing perspective by linking everything with God 'Allah' that He will take care of everything.

"All you have to do is care, and this is a type of help and if any patient gets well with our help it would be a blessing from Allah, living and dying is Allah's decision, we can only take care so take care and help others, you will be rewarded InshaAllah [if Allah wills] so we say all this to each other and we give support to each other". Service Provider, Dadu

"I was continuously in contact with her [COVID19 positive health worker] and tell her to take care of herself and she was telling her children whether test will come negative or not. She was scared that she had corona and she would die. Yes, we told her God will help you. We have doctors with us and our district manager and medical superintendent are with us, they were talking to her continuously. They were also consoling here that corona will end why you are worried?" Service Provider, Khairpur

"Reassuring, giving trust, and with positive thinking for the future, telling them that we are messengers, Allah has placed us to serve these people, to take care of them, to tell them preventions". Lady Health Supervisor, Rawalpindi

A few HCWs kept themselves busy in extra-curricular activity to deal with their stress like reading or even social media.

"I kept myself engaged because whenever I was free then I would get a lot stress when you hear news etc. I was studying and spent most of my time with my co-worker. And whenever I would go home, I would keep myself engaged mentally". Service Provider, Sialkot

For peer review only - http://bmjopen.bmj.com/site/about/guidelines.xhtml

2.4 Managerial support from supervisors

According to a majority of respondents, supervisors provided a lot of support in managing work-related issues that were causing stress. The supervisors primarily managed issues of their teams by themselves, or at times, by engaging a higher (district) administrative health authorities. Effective communication with team that included constant motivation and awareness raising was instrumental in addressing day-to-day challenges. At some instances, health managers also offered intermittent breaks to the health workers who were unwell or became exhausted due to work. A couple of respondents also mentioned that the health facility was closed for a few days when the COVID19 positive staff was quarantined.

"We fulfil their demands, if there is a salary issue, if they haven't received their salary from 2 to 3 months, we write it to DHO that they will not do work with interest so they should be given their salary on time and they forward it to upper department and their problems are solved. Service Provider, Dadu

"I counsel them that see this is difficult for us; you cooperate with me and I will cooperate with you. If you have any problem then you can tell me and take off. If there is no issue then take half day off but come to work next day. So I tried to make them relax like this". Health Manager, Karachi

3. Mental health needs of healthcare workers and recommended solutions

3.1 Safe and conducive work environment

Safe working environment was identified as one of the prominent needs of HCWs. It primarily meant timely provision of PPE kits that should include gloves, face-masks, and hand sanitizers, and provision of security especially to deal with aggressive patients.

"We should have received PPE kits in the beginning before [COVID19] peak time. When cases were increasing in China, since then we should have made ourselves prepared for this". Service Provider, Multan

"Security should be given especially in this COVID situation. Because when patients come to the health facility and the doctor fails to examine them then they create a scene or become aggressive, and misbehave". Service Provider, Sialkot

HCWs who were assigned additional responsibilities related to COVID19 expressed a dire need of intermittent breaks under certain circumstances – for example, when a worker is ill, exhausted due to increased work-load, or experiences any kind of stress.

"If someone appears COVID-19 positive then that person is placed in 14 days quarantine. So if he is already in a stressful condition, his mental health isn't able to hold so at least until he is normalized, there should be some relief given". Service Provider, Faisalabad

"They [health workers] say that how can we perform our normal duties and COVID ward both simultaneously? So they should be given holidays. Our demand is that our staff should be given more holidays and good packages and some financial bonuses should be provided". Health Manager, MirpurKhas

For peer review only - http://bmjopen.bmj.com/site/about/guidelines.xhtml

3.2 Awareness about evolving situation of COVID19 and corrective actions

A majority of health professional expressed the need for having updated information about the evolving situation regarding COVID19. They also identified the need of receiving timely guidelines about the corrective actions to deal with constantly changing situation.

"There should have been sessions about the forthcoming epidemic. Like this what is this disease, what are its consequences, how to deal with it. There should've been such learnings that they prepare their workers. They just said that 'wear masks', and what's being told on TV". Mealth Manager, Khairpur

3.3 Psychosocial support from specialized mental healthcare workers:

Even though social support emerged as a key response-measure used by HCWs to cope with the prevailing stress, getting psychosocial help from specialized mental health professional was still identified as a need and future recommendation by majority of the study participants. Various suggestions were given to have such support in-place for the HCWs, which included: a) having a full-time specialist based district or sub-district level to provide needs-based support, b) conduction of periodic sessions on mental health, c) mobile team of psychologist to provide periodic support of health staff; and d) making mental health component part of the COVID19 trainings.

"Mental health is a drawback here. There should be a proper person, who should, first of all, educate the staff, that how will you improve the mental health of the people. Then, some training should be arranged there, meaning, there should be a proper person sitting there, right, like some psychiatrist, or some people like these". Lady Health Supervisor, Rawalpindi

"In my opinion, CEO of our hospital should depute someone from DHQ like 3 sessions in a week so that they can give sessions to healthcare workers, perform counselling and try to know their inner problems due to which they are disturbed so that their problems can be solved. And provide them relief if it is related to salary or any other issue". Service Provider, Sargodha

Some HCWs suggest use of mobile application to provide information about mental health and well-being.

"They should conduct seminars and this is the era of multimedia everyone has mobile and there should be more content on multimedia and social media about mental health so that we become mentally strong and cater things properly. There should be seminars and sessions and government should launch an app regarding this so that we get information from it regarding mental and physical health and keep on updating it". Health Manager, Gujrat

On the other hand, healthcare workers expressed the need of capacity building to provide adequate psychosocial support to co-workers and to their patients, as needed. These trainings are also expected to help them guide general patients or community people to follow COVID19 SOPs.

"No, patients aren't believing, that's why psychiatrist is important and there should be training provided by the psychiatrist. Our staff should be provided psychological training, we should know what they [patients] are saying and what their opinions are so then we can handle them accordingly". Health Manager, MirpurKhas

For peer review only - http://bmjopen.bmj.com/site/about/guidelines.xhtml

3.4 Appreciation and recognition of their work in the pandemic crisis:

HCWs demanded recognition and appreciation for the additional work that they had to do during COVID19 crisis. This need was particularly highlighted by those who were given additional responsibilities like secondment at isolation centres, contact tracing, and COVID19 testing of suspected cases. This recognition was demanded in the form of monetary incentive for risking their lives, or a letter of appreciation by the health department, or a continuous encouragement from their administrative leadership.

"Lady Health Workers are already doing extra work, putting their lives in danger, should have gotten some extra benefits for this, even if they had gotten any certificate. Even if we get one praise, they want to sacrifice everything". Lady Health Supervisor, Rawalpindi

"I would say that government should facilitate the doctor, increase the allowance, give them a complete personal kit, if someone dies, government should support their family, so this should be on government level, so doctors also work motivated… I would want that government should give full support, financially and morally". Service Provider, Khairpur

Discussion:

The current body of existing literature revolved predominantly around the mental health and well-being of HCWs who are involved in provision of specialised COVID-related health services. It largely neglects the PHCWs who are the first point of contact for patients and are also involved in wide range of COVID19-related activities, which in turn can impact their mental health. Our study is unique that it explored the psychological impact of COVID19, health system response, and sought suggestion and recommendations from the HCWs to address the psychological needs of PHCWs in pandemic crisis.

Reportedly, constant fear, anxiety and stress of being infected and infecting others was immense at onset of COVID19 outbreak and during its peak time. This was primarily triggered by the lack of information about the virus and its management, false rumours regarding COVID19, media-hype, lack of availability of PPEs, and not being able to distinguish between COVID19 positive and negative patients. Evidence show that exaggeration by media tend to negatively affect the mental health of HCWs [40,41]. Similarly, lack of knowledge about COVID19 infection is likely to increase anxiety/depression among physicians[42] and inadequate precautionary measures in the workplace causes fear and anxiety among HCWs[43].

Another important concern causing distress and frustration among healthcare workers was non-compliance of COVID19 SOPs by incoming patients and their companions. According to service providers, low literacy in rural populations, lack of awareness and non-serious attitude towards COVID19 were the primary reasons for this non-adherence. This is consistent with another study from Pakistan that identified non-compliance of COVID19 SOPs as one of the reasons for stress and anxiety among HCWs[32]. Misconceptions and stigma attached to COVID19 also made it immensely challenging for HCWs for contact tracing and COVID19 testing on suspected patients. In extreme cases, people became aggressive and reacted violently to HCWs. Similar incidents have been reported in Pakistan and other countries [44,45]. Such incidents have been associated with work-related stress, burn out, and post-traumatic stress disorder, which in turn negatively affect job performance and quality of services [46].

For peer review only - http://bmjopen.bmj.com/site/about/guidelines.xhtml

The psychological impact of COVID19 tapered off over time among PHCWs. PHCWs attributed the reduction of stress and anxiety the support provided by the government in the form of awareness raising sessions regarding COVID19 and its preventive measures, and provision of PPE. More importantly, social support by co-workers (including supervisors) emerged a key factor that alleviated the level of stress and fear among healthcare workers at the beginning when it was very high. Social support by co-workers has also been reported as an effective response-mechanism to deal with prevalent mental health issues among HCWs COVID19 crisis [47,48]. Given its significance, fostering a systematic mechanism of social support within healthcare system has also been recommended to safeguard mental health of HCWs during the pandemic[48]. Consistent with other studies [9], patient visits at PHCWs significantly reduced due to the fear of COVID19 infections among general population. Work-load was only a concern of PHCWs were assigned additional task regarding COVID19, and these HCWs reported experiences of exhaustion due to additional responsibilities. They also demanded recognition and appreciation in the form official appreciation letter or monetary incentives for risking their lives. This finding is consistent with other studies where HCWs expressed similar needs [16,44].

In the end, PHCWs rendered few recommendations to address their psychological needs in pandemic crisis. Firstly, they expressed the necessity of having a safe and conducive working environment that included timely provision of PPE for all staff members, safety and security of HCWs to deal with difficult patients, and intermittent breaks – especially for those who are assigned additional COVID19-related responsibilities. The positive effect of safe and conductive work environment on mental health is already discussed above. Second recommendation was around preparedness of HCWs through continuous awareness raising and corrective actions about the evolving pandemic situation. Increased knowledge is also linked with reduced level of stress and anxiety among healthcare workers[42]. Thirdly, they highlighted the need for psychological counselling from mental health professionals and suggested different approach to fulfil this need. Call centres have been established in many countries – including Pakistan[49] - to provide needs-based psychological support to HCWs. In Pakistan, this programme perhaps need to expand their scope and provide more targeted services to the PHCWs. Additionally, HCWs requested the need for training to be able to provide psychosocial support to co-worker and dealing with difficult patients or people in the communities. There has already been a debate about potential role of community health workers in the provision of psychosocial support to people during pandemic crisis [50]. The existing "WeCare" programme of the Pakistani government could also be effectively integrated with PHC settings to assist HCWs in providing support to the incoming patients. Lastly, a key need identified by the HCWs, especially those who were assigned additional tasks related to COVID19, was appreciation and recognition for their work in pandemic crisis. They particularly stressed on developing a formal mechanism for the acknowledgement of their efforts via appreciation letter or in the form of monetary incentive or risk allowances. Similar need was also identified in other studies as well [51].

Strengths and Limitations:

In terms of strengthens, our study used gather experiences of a neglected group HCWs that belonged to PHC settings. It uses an inductive interpretative approach to achieve study objectives by gathering information from diverse group of service providers and health managers, including lady health workers. Importantly, this research revealed the perspective and experience of HCWs from the rural settings which are largely neglected in the current body of knowledge. It allowed researchers to look for converging and diverging lines of inquiry to identify common themes/ concepts and incongruence between data sources. On the contrary, a major limitation was that all interviews were conducted over phone which may have constrained rapport building with participant and obtain no-verbal cues during the interviews. In addition, the interviewees might have been distracted during interviews by the activities in their environment and might have

For peer review only - http://bmjopen.bmj.com/site/about/guidelines.xhtml

experienced fatigue due to long telephonic interviews. Another limitation was that the study was conducted with a focus on public sector hospitals in only two provinces of Pakistan (Sindh and Punjab), which would limit the transferability of the findings to other provinces[52]. However, this study may provide insights for similar PHC settings across Pakistan that are interested in understanding the psychological impact and mental health needs of health service providers during Covid-19. Lastly, the study was unable to carry out member checking with study participants, which may have affected the validity of study findings.

Conclusion:

Our study concludes that the stress and anxiety among primary healthcare workers was immense at the beginning of this outbreak or when the COVID19 transmission was at its peak. However, it tapered-off as they become more aware of the virus, receive PPEs, and number of COVID19 cases decreased in their community. Mental health of either health worker or patients were not covered in the trainings offered to them – neither had they had access to any professional psychological support. Our study identified a need for a comprehensive support that should be provided to primary healthcare workers in a timely manner, including informational support (e.g. continuous awareness about evolving situation and corrective actions); safe and conductive work environment (e.g. provision of personal protective equipment, personal security to deal with violent reaction of community people due to stigma); organisational support (e.g. intermittent breaks, appreciation and recognition in the form of appreciation letters or monetary incentives (especially for those who are assigned additional responsibilities); and frequent professional psychosocial support to cope with stress but also capacity building trainings on mental health that will enable them deal with difficult patients or community people.

Contributions:

WH and BIA conceptualised the study with inputs from BK, ZF and SS. WH, BIA, BK, ZF, and AF developed interview guide. HJ and MW facilitated in conducting the study. WH and AF performed data analysis. WH took the lead in writing the manuscript. All authors critically reviewed the manuscript and provided feedback in shaping the write-up, analysis and presentation of results.

Conflicts of interest: None

Funding: This work was co-funded by the Aga Khan University, Pakistan and the World Health Organization

Data sharing: The data will be available upon request

Ethics: Ethics approval was obtained from Aga Khan University (Ref. number: 2020-5186-11602) and National Bioethics Committee of Pakistan (Ref. Number: 4-87/COVID-45/NBC/20/393).

Acknowledgements: We are grateful to our implementing partners: Department of Health, Government of Sindh; Primary and Secondary Healthcare Department (P&SHD) and Specialized Healthcare & Medical Education Department from Government of Punjab for extending their full their support and guidance to conduct this survey. We are also thankful to all our study participants for sparing their precious time and made themselves available for the interviews. Last but not the least, we thank our data collectors and project management team: Zafar Dehraj, Imran Sheikh,

For peer review only - http://bmjopen.bmj.com/site/about/guidelines.xhtml

Sajid Brohi, Noreen Afzal and Ghani Muhammad who managed the implementation of this study in a proficient manner during the pandemic.

Disclaimer:

The views expressed in this manuscript are those of the authors and contributors, and do not necessarily reflect those of the World Health Organization, or the organization to which the authors are affiliated.

For peer review only

For peer review only - http://bmjopen.bmj.com/site/about/guidelines.xhtml

Reference List

1. Li Q, Guan X, Wu P*, et al.* Early Transmission Dynamics in Wuhan, China, of Novel CoronavirusΓÇôInfected Pneumonia. *New England Journal of Medicine* 2020; 382:1199-1207.https://doi.org/10.1056/NEJMoa2001316

2. Center for Systems Science and Engineering. Coronavirus Resource Center. 27-4-2020. Baltimore, United States, Johns Hopkins University. 27-4-2020.
Ref Type: Online Source

3. Castella TD. Nursing Times. 2021. EMAP. 20-9-2021.
Ref Type: Online Source

4. Wu P, Fang Y, Guan Z*, et al.* The Psychological Impact of the SARS Epidemic on Hospital Employees in China: Exposure, Risk Perception, and Altruistic Acceptance of Risk. *Can J Psychiatry* 2009; 54:302-311.https://doi.org/10.1177/070674370905400504

5. Li Y, Scherer N, Felix L*, et al.* Prevalence of depression, anxiety and post-traumatic stress disorder in health care workers during the COVID-19 pandemic: A systematic review and meta-analysis. *PLoS ONE* 2021; 16:e0246454.https://doi.org/10.1371/journal.pone.0246454

6. Moitra M, Rahman M, Collins PY*, et al.* Mental Health Consequences for Healthcare Workers During the COVID-19 Pandemic: A Scoping Review to Draw Lessons for LMICs. *Frontiers in Psychiatry* 2021; 12:22.https://www.frontiersin.org/article/10.3389/fpsyt.2021.602614

7. Vizheh M, Qorbani M, Arzaghi SM*, et al.* The mental health of healthcare workers in the COVID-19 pandemic: A systematic review. *Journal of Diabetes & Metabolic Disorders* 2020; 19:1967-1978.https://dx.doi.org/10.1007%2Fs40200-020-00643-9

8. Dunlop C, Howe A, Li D*, et al.* The coronavirus outbreak: the central role of primary care in emergency preparedness and response. *BJGP Open* 2020; 4:bjgpopen20X101041.https://pubmed.ncbi.nlm.nih.gov/31992543

9. Rawaf S, Allen LN, Stigler FL*, et al.* Lessons on the COVID-19 pandemic, for and by primary care professionals worldwide. *European Journal of General Practice* 2020; 26:129-133.https://doi.org/10.1080/13814788.2020.1820479

10. Sarti TD, Lazarini WS, Fontenelle LF*, et al.* Qual o papel da Aten+º+úo Prim+íria +á Sa+¦de diante da pandemia provocada pela COVID-19? *Epidemiologia e Serviços de Saúde* 2020; 29.https://doi.org/10.5123/s1679-49742020000200024

11. Bhaumik S, Moola S, Tyagi J*, et al.* Community health workers for pandemic response: a rapid evidence synthesis. *BMJ Global Health* 2020; 5:e002769.http://gh.bmj.com/content/5/6/e002769.abstract

For peer review only - http://bmjopen.bmj.com/site/about/guidelines.xhtml

12. Gray DM, II, Joseph JJ, Olayiwola JN. Strategies for Digital Care of Vulnerable Patients in a COVID-19 World–Keeping in Touch. *JAMA Health Forum* 2020; 1:e200734.https://doi.org/10.1001/jamahealthforum.2020.0734

13. First in, last out - The role of family doctors in the fight against novel coronavirus. 2020. World Organization of Family Doctors, WONCA. 7-10-2021.
Ref Type: Online Source

14. Mash B. Primary care management of the coronavirus (COVID-19). *S Afr Fam Pract (2004)* 2020; 62:e1-e4.https://pubmed.ncbi.nlm.nih.gov/32242438

15. Waters R. Community Workers Lend Human Connection To COVID-19 Response. *Health Aff (Millwood)* 2020; 39:1112-1117.https://doi.org/10.1377/hlthaff.2020.00836

16. Strengthening the frontline: How primary health care helpshealth systems adapt during the COVID-19 pandemic. 10-2-2021. OECD Policy Responses to Coronavirus (COVID-19). 12-5-2021.
Ref Type: Report

17. National Center for Immunization and Respiratory Diseases (NCIRD) Division of Viral Diseases. Engaging Community Health Workers to Support Home-based Care for People with COVID-19 in Low-Resource Non-U.S. Settings. 7-4-2021. United States, Center for Disease Control and Prevention. 12-5-2021.
Ref Type: Online Source

18. De Kock JH, Latham HA, Leslie SJ*, et al.* A rapid review of the impact of COVID-19 on the mental health of healthcare workers: implications for supporting psychological well-being. *BMC Public Health* 2021; 21:104.https://doi.org/10.1186/s12889-020-10070-3

19. Tremblay S, Castiglione S, Audet LA*, et al.* Conducting Qualitative Research to Respond to COVID-19 Challenges: Reflections for the Present and Beyond. *International Journal of Qualitative Methods* 2021; 20:16094069211009679.https://doi.org/10.1177/16094069211009679

20. Zace D, Hoxhaj I, Orfino A*, et al.* Interventions to address mental health issues in healthcare workers during infectious disease outbreaks: A systematic review. *J Psychiatr Res* 2021; 136:319-333.https://www.sciencedirect.com/science/article/pii/S0022395621000868

21. Torjesen I. Covid-19 will become endemic but with decreased potency over time, scientists believe. *BMJ* 2021; 372:n494.http://www.bmj.com/content/372/bmj.n494.abstract

22. Phillips N. The coronavirus is here to stay — here's what that means. *Nature* 2021; 590:382-384.https://doi.org/10.1038/d41586-021-00396-2

23. Gunnell D, Appleby L, Arensman E*, et al.* Suicide risk and prevention during the COVID-19 pandemic. *The Lancet Psychiatry* 2020.https://doi.org/10.1016/S2215-0366(20)30171-1

24. Population Reference Bureau. 2021 World Population Data Sheet. 2021. Washington DC, Population Reference Bureau. 28-9-2021.
Ref Type: Online Source

For peer review only - http://bmjopen.bmj.com/site/about/guidelines.xhtml

25. National Information Technology Board. COVID-19 Dashboard. 27-4-2020. Islamabad, Government of Pakistan. 27-4-2020.
Ref Type: Online Source

26. Hassan A, Mahmood K, Bukhsh HA. Healthcare System Of Pakistan. *International Journal of Advanced Research and Publication* 2017; 1:170-173.https://www.ijarp.org/published-research-papers/oct2017/Healthcare-System-Of-Pakistan.pdf

27. Waris A, Atta UK, Ali M*, et al.* COVID-19 outbreak: current scenario of Pakistan. *New Microbes and New Infections* 2020; 35:100681.http://www.sciencedirect.com/science/article/pii/S2052297520300330

28. Khalid A, Ali S. COVID-19 and its Challenges for the Healthcare System in Pakistan. *Asian Bioethics Review* 2020; 12:551-564.https://dx.doi.org/10.1007%2Fs41649-020-00139-x

29. Fullman N, Yearwood J, Abay SM*, et al.* Measuring performance on the Healthcare Access and Quality Index for 195 countries and territories and selected subnational locations: a systematic analysis from the Global Burden of Disease Study 2016. *Lancet* 2018; 391:2236-2271.https://doi.org/10.1016/S0140-6736(18)30994-2

30. Abdullah MA, Mukhtar F, Wazir S*, et al.* The Health Workforce Crisis in Pakistan: A Critical Review and the Way Forward. *World Health & Population* 2014; 15:4-12.https://www.longwoods.com/product/24031

31. Asim SS, Ghani S, Ahmed M*, et al.* Assessing Mental Health of Women Living in Karachi During the Covid-19 Pandemic. *Frontiers in Global Women's Health* 2021; 1:594970.https://doi.org/10.3389/fgwh.2020.594970

32. Sandesh R, Shahid W, Dev K*, et al.* Impact of COVID-19 on the Mental Health of Healthcare Professionals in Pakistan. *Cureus* 2020; 12:e8974.https://pubmed.ncbi.nlm.nih.gov/32775056

33. Arshad MS, Hussain I, Nafees M*, et al.* Assessing the Impact of COVID-19 on the Mental Health of Healthcare Workers in Three Metropolitan Cities of Pakistan. *Psychol Res Behav Manag* 2020; 13:1047-1055.https://pubmed.ncbi.nlm.nih.gov/33244279

34. Government of Punjab. National Programme for Family Planning & Primary Health Care, Pakistan. 2011. National Programme for Family Planning & Primary Health Care. 4-5-2015.
Ref Type: Online Source

35. Creswell JW, Poth CN. Qualitative Inquiry and Research Design: Choosing Among Five Approaches. Fourth Edition ed. SAGE Publishing; 2017.

36. Yin RK. Case Study Research and Applications: Design and Methods. Sixth Edition ed. SAGE Publishing; 2017.

37. Fusch PI, Ness LR. Are We There Yet? Data Satur et? Data Saturation in Qualitative Research. *The Qualitative Report* 2015; 20:1408-1416.https://nsuworks.nova.edu/cgi/viewcontent.cgi?article=2281&context=tqr

For peer review only - http://bmjopen.bmj.com/site/about/guidelines.xhtml

38. NVivo qualitative data analysis software [QSR International Pty Ltd.; 2015.

39. Tong A, Sainsbury P, Craig J. Consolidated criteria for reporting qualitative research (COREQ): a 32-item checklist for interviews and focus groups. *Int J Qual Health Care* 2007; 19:349-357.https://doi.org/10.1093/intqhc/mzm042

40. Su Z, McDonnell D, Wen J*, et al.* Mental health consequences of COVID-19 media coverage: the need for effective crisis communication practices. *Globalization and Health* 2021; 17:4.https://doi.org/10.1186/s12992-020-00654-4

41. World Health Organization. Health workforce policy and management in the context of the COVID-19 pandemic response: interim guidance, 3 December 2020. 3-12-2020. Geneva, World Health Organization. 10-10-2021.
Ref Type: Report

42. Amin F, Sharif S, Saeed R*, et al.* COVID-19 pandemic- knowledge, perception, anxiety and depression among frontline doctors of Pakistan. *BMC Psychiatry* 2020; 20:459.https://doi.org/10.1186/s12888-020-02864-x

43. Hoernke K, Djellouli N, Andrews L*, et al.* Frontline healthcare workers' experiences with personal protective equipment during the COVID-19 pandemic in the UK: a rapid qualitative appraisal. *BMJ Open* 2021; 11:e046199.http://bmjopen.bmj.com/content/11/1/e046199.abstract

44. Martins RS, Bhatti OA, Mian AI. Violence Against Health Care Workers in Pakistan During the COVID-19 Pandemic. *JAMA Health Forum* 2020; 1:e201263.https://doi.org/10.1001/jamahealthforum.2020.1263

45. Bhatti OA, Rauf H, Aziz N*, et al.* Violence against Healthcare Workers during the COVID-19 Pandemic: A Review of Incidents from a Lower-Middle-Income Country. *Ann Glob Health* 2021; 87:41.https://pubmed.ncbi.nlm.nih.gov/33977084

46. Shaikh S, Baig LA, Hashmi I*, et al.* The magnitude and determinants of violence against healthcare workers in Pakistan. *BMJ Global Health* 2020; 5:e002112.http://gh.bmj.com/content/5/4/e002112.abstract

47. Al-Mansour K, Alfuzan A, Alsarheed D*, et al.* Work-Related Challenges among Primary Health Centers Workers during COVID-19 in Saudi Arabia. *Int J Environ Res Public Health* 2021; 18:1898.https://pubmed.ncbi.nlm.nih.gov/33669440

48. Labrague LJ. Psychological resilience, coping behaviours and social support among health care workers during the COVID-19 pandemic: A systematic review of quantitative studies. *J Nurs Manag* 2021; n/a.https://doi.org/10.1111/jonm.13336

49. Ministry of National Health Services Regulation and Coordination (MoNHSRC). We CARE. 2020. Islamabad, Government of Pakistan. 18-9-2021.
Ref Type: Online Source

50. Mistry SK, Harris-Roxas B, Yadav UN*, et al.* Community Health Workers Can Provide Psychosocial Support to the People During COVID-19 and Beyond in Low- and Middle- Income Countries.

For peer review only - http://bmjopen.bmj.com/site/about/guidelines.xhtml

Frontiers in Public Health 2021; 9:800.https://www.frontiersin.org/article/10.3389/fpubh.2021.666753

51. Shaukat N, Ali DM, Razzak J. Physical and mental health impacts of COVID-19 on healthcare workers: a scoping review. *Int J Emerg Med* 2020; 13:40.https://pubmed.ncbi.nlm.nih.gov/32689925

52. Donmoyer R. Generalizability and the Single-Case Study. In: Gomm R, Hammersley M, Foster P, editors. Case Study Method. SAGE publications Ltd.; 2009.

For peer review only

For peer review only - http://bmjopen.bmj.com/site/about/guidelines.xhtml

BMJ Open

Impact of COVID19 on mental health of primary healthcare workers in Pakistan: lessons from a qualitative inquiry

Journal:	*BMJ Open*
Manuscript ID	bmjopen-2022-065941.R1
Article Type:	Original research
Date Submitted by the Author:	24-Oct-2022

Complete List of Authors:	Hameed, Waqas; The Aga Khan University, Community Health Sciences<br>Avan, Bilal Iqbal; London School of Hygiene and Tropical Medicine,<br>Feroz, Anam Shahil; Aga Khan University, Department of Community Health Sciences<br>Khan, Bushra; University of Karachi, Department of Psychology<br>Fatmi, Zafar; Aga Khan University, Department of Community Health Sciences<br>Jafri, Hussain; Fatima Jinnah Medical University<br>Wassan, Mansoor Ali; Government of Sindh<br>Siddiqi, Sameen; Aga Khan University Medical College Pakistan, Community Health Sciences Department

**Primary Subject Heading**:	Mental health
Secondary Subject Heading:	Mental health, Public health, Qualitative research
Keywords:	MENTAL HEALTH, HEALTH SERVICES ADMINISTRATION & MANAGEMENT, COVID-19

SCHOLARONE™
Manuscripts

I, the Submitting Author has the right to grant and does grant on behalf of all authors of the Work (as defined in the below author licence), an exclusive licence and/or a non-exclusive licence for contributions from authors who are: i) UK Crown employees; ii) where BMJ has agreed a CC-BY licence shall apply, and/or iii) in accordance with the terms applicable for US Federal Government officers or employees acting as part of their official duties; on a worldwide, perpetual, irrevocable, royalty-free basis to BMJ Publishing Group Ltd ("BMJ") its licensees and where the relevant Journal is co-owned by BMJ to the co-owners of the Journal, to publish the Work in this journal and any other BMJ products and to exploit all rights, as set out in our licence.

The Submitting Author accepts and understands that any supply made under these terms is made by BMJ to the Submitting Author unless you are acting as an employee on behalf of your employer or a postgraduate student of an affiliated institution which is paying any applicable article publishing charge ("APC") for Open Access articles. Where the Submitting Author wishes to make the Work available on an Open Access basis (and intends to pay the relevant APC), the terms of reuse of such Open Access shall be governed by a Creative Commons licence – details of these licences and which Creative Commons licence will apply to this Work are set out in our licence referred to above.

Other than as permitted in any relevant BMJ Author's Self Archiving Policies, I confirm this Work has not been accepted for publication elsewhere, is not being considered for publication elsewhere and does not duplicate material already published. I confirm all authors consent to publication of this Work and authorise the granting of this licence.

For peer review only

Bismillah hir Rahman nir Raheem

Title:
Impact of COVID19 on mental health of primary healthcare workers in Pakistan: lessons from a qualitative inquiry

Short title: COVID19 and mental health of primary healthcare workers

Authors:
Waqas Hameed[1], Bilal Iqbal Avan[2], Anam Shahil Feroz[1], Bushra Khan[3], Zafar Fatmi[1], Hussain Jafri[4], Mansoor Ali Wassan[5], Sameen Siddiqi[1]

[1]Department of Community Health Sciences, Aga Khan University, Karachi, Pakistan
[2]Department of Clinical Research, London School of Hygiene and Tropical Medicine, London, United Kingdom
[3]Department of Psychology, University of Karachi, Sindh, Pakistan
[4]Fatima Jinnah Medical University, Lahore, Pakistan
[5]Department of Health, Government of Sindh, Pakistan

Correspondence:
Bilal Iqbal Avan
Associate Professor
Department of Population Health
London School of Hygiene and Tropical Medicine
Keppel St, London WC1E 7HT, United Kingdom
bilal.avan@lshtm.ac.uk

For peer review only

For peer review only - http://bmjopen.bmj.com/site/about/guidelines.xhtml

Abstract

Objectives
The existing literature regarding the mental health consequences of COVID19 among healthcare workers resolves predominately around specialised hospital settings, while neglecting primary healthcare workers who are the first point of contact for patients. In view of negligible evidence, this study explored mental health impact of COVID19, health system response, and sought suggestions and recommendations from the primary healthcare workers (PHCWs) to address their mental health needs in pandemic crisis.

Design
We employed a qualitative exploratory design.

Setting: A total of 42 primary healthcare facilities across 15 districts in Sindh and Punjab provinces of Pakistan.

Participants: We telephonically conducted 47 in-depth interviews with health service providers and hospital managers. A combination of inductive and deductive approach was used for data analyses using NVivo version 11.0.

Results
There was immense fear, stress and anxiety among PHCWs of being infected and infecting their families at the beginning of this outbreak and its peak which tapered off over time. It was triggered by lack of information about the virus and its management, false rumours, media-hype, lack of personal protective measures (PPEs), non-cooperation from patients and community people. Awareness raising trainings and PPEs provided by the healthcare system, while emotional support from co-workers and supervisors was instrumental in addressing their mental health needs. Additionally, they recommended appreciation and recognition, and provision of psychosocial support from mental health professionals.

Conclusion
Primary healthcare system should be prepared to provide timely informational (e.g. continuous updates in training and guidelines), instrumental (e.g. provision of personal protective equipment, appreciation and recognition), organisational (e.g. safe and conducive work environment), and emotional and psychosocial support (e.g. frequent or needs-based session from mental health professionals) to PHCWs in order to mitigate the mental health impact of pandemic crisis.

Keywords: COVID19, primary healthcare, mental health, psychosocial, health system

For peer review only - http://bmjopen.bmj.com/site/about/guidelines.xhtml

Strengths and limitations of this study:

- Our study gathered mental health experiences of a neglected group health care workers that belonged to primary healthcare settings
- Unlike urban-focused studies, the participants in this study were invited across fifteen urban and rural districts in two most populous provinces of Pakistan
- Conduction of phone-based interviews due to the restricted mobility during COVID-19 was a key study limitation which may introduce some risk of bias as the interviewers did not have the opportunity to build rapport as in the case of face-to-face interviews

For peer review only

For peer review only - http://bmjopen.bmj.com/site/about/guidelines.xhtml

Introduction:

The novel coronavirus disease 2019 (COVID19) has swept across the world.[1] As of September 28, 2021, 232 million people have been infected by COVID19 and approximately 4.75 million people have lost their lives worldwide[2], including 115,000 healthcare workers (HCWs)[3]. This large-scale unprecedented public health catastrophe, placed the frontline HCWs, who are directly involved with all aspects of care of COVID19 patients, at risk of developing psychological distress and other mental health symptoms.[4] Studies conducted thus far provide evidence of the adverse impact of COVID19 on mental health and well-being of HCWs, primarily those who are involved in providing specialised care to COVID19 patients[5]. According to a systematic review, the pooled prevalence of depression, anxiety and post-traumatic stress disorder among HCWs related to the COVID19 pandemic across 21 (low to high income) countries hovered around 22%[5]. The prevalence of such mental health disorders vary according to sex, age, and role of HCWs[6,7].

While these studies made substantial contribution to inform strategies in alleviating the burden of psychological distress among HCWs, the prime focus has been on professionals who are working in hospital settings and are primarily engaged in the provision of specialised care to COVID19 patients[6,7]. In contrast, primary healthcare workers (PHCWs) have been managing a major share of the burden of COVID19 related care[8] by being the first point of contact for a broad spectrum of COVID19 patients, whether suspected, confirmed (with or without symptoms), or exposed to case(s), as well as those with non-COVID19 health needs[9]. Studies indicate that some 80% of cases are mild and the majority of moderate cases seek primary healthcare (PHC) services as the entry point for getting medical care[10].

Community health workers (CHWs) also played a distinct role during COVID19, in lower middle-income countries (LMICs), by being involved in community awareness, engagement and sensitisation and contact tracing though at the cost of routine service delivery [11]. These newly assigned tasks during pandemic not only exposed them to increased risk of infection, but they also experienced stigmatisation, isolation or social ostracisation[11]. Ironically, they reportedly received inadequate support from health system in the form of personal protective equipment[12], and logistics and supportive supervision. Besides that, PHCWs are also responsible to manage the aftermath of contingencies and bearing the collateral damage caused by reallocating resources to COVID19 whilst inadvertently hindering access to a wide range of primary healthcare (PHC)services[13,14.] This put them under stressful situations during this pandemic[15]. A study from Canada showed that interpersonal care team rapidly shifted their practices to support their patients during COVID19 pandemic; and they experience a surge in mental health issues among their patients[16]. Similarly, a large-scale survey from Canada revealed that COVID19 resulted in increased work-load, loss of employment, redeployment to new setting, concerns for health and safety among social workers at the frontline[17].

Despite the ongoing debate on how strengthening PHC (including CHWs) can help health systems adapt during the COVID19 pandemic[18,19] the significance and instrumental role of PHCWs in the times of COVID19 pandemic has received little attention of the global research community. A large majority of the research that assessed mental health consequences of COVID19 among HCWs has focused in the specialised hospital settings[20]. Furthermore, there are few qualitative studies that provide in-depth mental health experiences of HCWs, in general, as well as those working in PHC settings [21]. Even a recent effort to consolidate potential interventions to address mental health issues of HCWs during such outbreak is largely based evidence generated from HCWs working in specialised settings, while neglecting the mental health needs of and solutions for PHCWs[22].

For peer review only - http://bmjopen.bmj.com/site/about/guidelines.xhtml

Keeping in view of the role that PHCWs as alluded to above, we hypothesised that they may have suffered from similar mental health issues as faced by HCWs working in specialised healthcare settings. Furthermore, as COVID19 is expected to become endemic[23,24], the role of PHC becomes pivotal for its community-based information structure to identify risk groups, monitor compliance, provide preventive and promotional services, and contact tracing in this and similar future pandemic. Hence, their mental health needs, readiness and response become central to tackling such issues. In order to better support PHCWs with their adapted role during COVID19, interventions are needed that could effectively cater to their mental health (which include mental, emotional and social) needs in the long run [25]. With this rationale, we set following research questions for this research that focuses on public-sector PHCWs in Pakistan.

Research questions:

1. What is the perceived mental health impact of COVID19 on PHCWs in Pakistan?
2. How health system has responded to the mental health needs of PHCWs?
3. What are the recommendations to support mental health and well-being of PHCWs in pandemic crisis?

Country Context:
Pakistan has a population of over 225 million inhabitants[26]. The first COVID19 case was identified in February 2020, and since then a total number of confirmed cases exceed 1.2 million along with 27,638 deaths has been registered (till September 28, 2021)[27]. The country has experienced four waves – each with different variant including delta, more recently[27]. With a focus on vulnerable groups of HCWs and elderly (65+ years) people, the country embarked on a nation-wide vaccine campaign for COVID19. The vaccine is now available for everyone except for children under the age of 12 years[27]. While several vaccines are safe and effective, there is strong emphasis on practicing preventive measures by the government.

Public-sector healthcare in Pakistan is delivered through a three-tiered health system. The PHC provides services through a community-based component comprising over 100,000 CHWs and health facility component that includes almost 10,000 basic health units (BHUs), rural health centres (RHCs), maternal and child centres (MCH), and civil dispensaries[28]. On average, a BHU and RHC covers a population of 25,000 and 80,000, respectively. The government of Pakistan has taken several measures to combat this deadly pneumonia virus[29] but it leaves a lot to be desired[30]. The health system of Pakistan is weak[31] and with critical shortage of health workforce, and is not prepared to sustain a major surge in COVID-19 cases[32]. Few studies have been conducted to assess the impact of COVID19 on mental health of HCWs; while these studies do identify symptoms of mental disorders, all have focused on tertiary level of health system [33-35].

Methods:

Study design and setting
A qualitative exploratory design was adopted using in-depth interview technique. Exploratory design enabled us to gather detailed description of HCWs experiences during the COVID19 pandemic. The study was conducted in 42 health facilities across 15 districts of Sindh and Punjab provinces of Pakistan. Districts where the cumulative number of COVID19 cases were high since the onset of this outbreak till date of survey were selected. Participants were selected using a criterion purposive sampling approach from randomly selected public health facilities stratified by districts and type of health facilities (BHUs and RHCs). We also collected contact details of affiliated community health workers (referred to as “Lady Health Workers”[36] hereafter) from the in-charge of the selected health facility.

Recruitment of study participants:

For peer review only - http://bmjopen.bmj.com/site/about/guidelines.xhtml

The study participants were classified into three groups: lady health workers (LHWs), facility-based health service providers (doctor, nurse, midwife, lady health visitor), and administrators/managers (medical superintendent, lady health supervisors). Lady health workers are attached to a local health facility, but they are primarily community-based, working from their homes. They serve the whole community, but they play a particularly important role in maternal and child health care in rural and urban slum communities by coordinating efforts with traditional birth attendants and midwives[37]. In contrast, the facility-based healthcare workers are based at PHC facilities and they provide following services: general out-patient services, antenatal care, deliveries, postnatal care, and vaccination. In addition of these, diagnostic services (lab, x-ray) and dental outpatient services are also provided in RHCs. The PHCWs were primarily involved in awareness raising and contact training regarding COVID19. Some of the facility-based staff were also seconded to COVID19 isolation centres. Even though administrators at the PHC also often directly involved in service provision, we kept this distinction to see any diversity in their responses as opposed to their counterparts. The study participants were recruited after obtaining formal permission from the higher government authorities, by contacting administrators of the randomly selected health facility.

Data collection:

A standard semi-structured guide was used to interview all study participants, which was developed following extensive review of literature and consultation. It comprised of three major themes: a) Impact of COVID-19 on mental health and well-being of health care professionals; b) Mechanism available within healthcare system to address mental health needs; and c) Mental health needs of PHCWs, and suggestions and recommendations to address their needs. Open-ended questions and probes encouraged free flow of information from participants to developed deeper understanding of their experiences. The interview guide was thoroughly reviewed and discussed by the core research investigators (WH, BIA, ZF, AF, NA, BK and SSQ).It was then pre-tested and revised based on few PHCWs in districts that were included in the larger quantitative survey[38,39,40]. During pre-testing, no major current challenges were reported by most PHCWs since the first wave has already passed[40]. Therefore, the guideline was revised to gather their experiences from the onset of COVID19 to current situation, and getting insights on how situation evolved over time. (see supplementary file 1)

The data collection took placed between August and October 2020. Owing to restricted mobility due to COVID19, a total of 47 in-depth interview (IDIs) (Sindh: 29; Punjab: 18) were conducted telephonically until data saturation was achieved[41]. In the first phone call, participant was informed about the study and scheduled an appropriate data and time for the interview, if the participant is busy. Subsequent call was made at the schedule time for the interview. Experienced data collectors with a background in sociology or psychology were trained to conduct the interviews in local language (Urdu). All the calls were made from the Aga Khan University under close supervision of study investigators. Data analysis was concurrently performed to determine the point of saturation. On average, the interviews lasted for 25-35 minutes. With the consent of participants, all the interviews were audio-recorded on tablets using a built-in call recording feature in the tablets. It was ensure that participant is comfortable during the interview and no one is sitting near to them whose presence could possibly influence his/her responses. Data collectors were also encouraged to maintain key interview notes. A linguistic expert later transcribed the recordings into English for analysis. The transcripts were not returned to the participants due to their busy schedule. Five participants refused to participate in the study; they were mainly the health managers due to their busy schedule. No repeat interview was conducted. To ensure data

For peer review only - http://bmjopen.bmj.com/site/about/guidelines.xhtml

quality, a few initial interviews were conducted by WH (PhD scholar – health system) and AF (PhD scholar – public health) in presence of trained data collectors. Both the authors were working as a university faculty at the time of the study and had vast experience of conducting such interviews.

Data analysis:
All interview notes, recordings, and transcriptions were imported into the NVivo version 11.0 software for analyses, with no identifying characteristics included in the transcriptions[42]. We explored divergent and congruent views of three groups, that is, facility-based service providers, administrators, and lady health workers. Conventional content analysis technique was used to inductively analyse all data[43]. Two independent researchers (WH and ASF) applied inductive coding while reviewing the transcriptions in NVivo[42] and identified new emerging codes and categories. The codes and themes were compared and the any discrepancies in the interpretation of codes and themes were discussed among the investigation team to reach a consensus. The discrepancies were discussed in the large team meeting until agreement on the themes was achieved. In situations when it was difficult to achieve consensus among the team, senior author facilitated discussions and helped obtain agreements among the research team members. In order to gain a more comprehensive understanding of mental health impact, health system response, and suggestions and recommendation to support their mental health needs, sub-themes were reviewed and compared by additional reviewers (BK and ZF) to examine corroboration and convergence. Independent review of data and coding allowed coherent interpretations that are grounded in the data[39] We did not obtain feedback from the participants on study findings.

Patient and public involvement
We sought inputs from target audience through pre-testing of interview guides to ensure comprehension of the questions. Furthermore, in consultation with provincial health departments we selected districts where COVID-19 cases were high. The focal person in each selected district was oriented about the study who connected our team with in-charge of health facility to avoid any miscommunication. Lastly, they also advised about the dissemination of research findings – that is, they specifically emphasis to share it through research briefs to reach wider audience in the country.

Ethical considerations
Due to the telephonic interview, verbal informed consent was taken from all study participants which included permission to audio-record their interviews and use anonymized quotes. All study participants were informed about the study purpose, their rights as study participants, confidentiality, and publication of study findings in anonymised manner. We shared official permission letters with study participants issued by respective health department of government of Punjab and Sindh provinces to build trust. Ethics approval was obtained from Aga Khan University (Ref. number: 2020-5186-11602) and National Bioethics Committee of Pakistan (Ref. Number: 4-87/COVID-45/NBC/20/393).

Consolidated criteria for reporting qualitative research (COREQ) guidelines were used to organise this paper[44]. (see supplementary file 2)

Results:
A total of 47 IDIs were included in the analysis. Table 1 shows the socio-demographic characteristics of participants. The mean age was 43.2 (±10.9) years and had an experience of

For peer review only - http://bmjopen.bmj.com/site/about/guidelines.xhtml

14.4 (±9.4) years. Approximately half were female (44.7%), 55.3% were health managers, and about 51.1% were working at or affiliated with the Basic Health Unit. The in-charge of health facilities (BHU and RHC) were all medical doctors and mostly males.

Table 1: Characteristics of study participants

Characteristics	n (%)
**Sex**	
Female	21 (44.7)
Male	26 (55.3)
**Age in years**	
Mean (±SD)	43.2 (10.9)
**Designation**	
Health service providers (doctor, nurse/midwife)	16 (34.0)
Health managers (lady health supervisors – supervises LHW, in-charge of health facility)	26 (55.3)
Lady Health Workers (LHWs)	5 (10.6)
**Type of health facility**	
Basic Health Unit (BHU)	24 (51.1)
Rural Health Centre (RHC)	23 (48.9)
**Total experience in years**	
Mean (±SD)	14.4 (9.4)

The main study findings are organised into three main themes: perceived mental health impact of COVID-19; support mechanisms to address their mental health needs; and suggestions recommendations made by PHCWs. Where observed, we also presented contrasting views of service providers and health managers, highlighting the diversity in provincial perspective (table 2).

Table 2: Themes and Categories

Themes	Categories
Theme 1: Perceived mental health impact of COVID19 on healthcare workers	1.1 Anxiety due to uncertainty of Covid-19 and media exaggeration<br>1.2 Fear of infection, isolation and stigma<br>1.3 Challenges regarding patients<br>1.4 Stress due to lack of availability of personal protective equipment<br>1.5 Stress due to excessive work-load
Theme 2: Available support mechanisms to address mental health needs of healthcare workers	2.1 Awareness raising of healthcare workers<br>2.2 Provision of personal protective equipment<br>2.3 Social support from co-workers and family members<br>2.4 Managerial support from supervisors
Theme 3: Mental health needs of healthcare workers and recommended solutions	3.1 Safe and conducive work environment<br>3.2 Awareness about evolving situation and corrective actions<br>3.3 Psychosocial support from mental health professionals<br>3.4 Appreciation and recognition of their work in the pandemic crisis

1. Mental health impact of COVID19 on healthcare workers

1.1 Anxiety due to uncertainty of COVID19 and media exaggeration

For peer review only - http://bmjopen.bmj.com/site/about/guidelines.xhtml

All study participants reported experiences of immense fear and anxiety due to the uncertainties around COVID19 at the onset of this outbreak in Pakistan. It was primarily triggered by lack of knowledge, prevalent misconceptions about COVID19, and being aware of its disastrous effect in the developed world. There was a perception that it was a fatal virus and whoever got it would die. Others were sceptical about its existence like how long will this outbreak last and will they remain bound forever to the new operational changes in service delivery.

"At the beginning, the real fear among people was that - a rumour spread that, if God Forbids, this much dangerous disease, and someone gets it he will die". Health manager, Ghotki.

"There's a bit of worriedness, mental pressure, what to do what not to do, how to go, should we go to their house or not, these problems happens, mental stress occurs" Lady Health Worker, Jamshoro".

For many HCWs was not being able to distinguish between COVID-19 positive and negative people around them due to its asymptomatic characteristics and lack of testing facility was mentally disturbing. It made the HCWs reluctant to interact with patients and community members, and even with their colleagues at the very beginning of the outbreak. This was consistent among facility-based service providers, health managers and lady health workers.

"We ourselves were also mentally disturbed, that how do we know, we are going on duties, on fields, we staffs are meeting each other so how do we know that who has corona symptoms and who does not". Lady Health Supervisor, Rawalpindi

1.2 Fear of infection, isolation and stigma

While most of the PHCWs were not directly involved in providing care to COVID19 patients, there was still some fear of being contracted with COVID19 during their interaction with patients at the beginning and during peak time. Similar fear existed among LHWs during their routine household and targeted visits for contact tracing.

"We were afraid that we might get infected or our families might get infected. So we spend all this time under stress. We were seeing that there were many positive cases in our neighbourhood and the news on media". Health Manager, Shaheed Benazirabad

Some of the facility-based staff was seconded to COVID19 isolation centres. It was highlighted that the level of fear was considerably higher among them. Similar, the level of fear possible transmission was higher among HCWs who had any chronic disease (e.g. diabetes) or had old parents or young children, be they work at the health facility or in the community.
"At that time, my female health workers were very disturbed mentally. She was like we have small kids how we will run out-patient services… how everything will be managed. Their families were also tensed that you are going on duties and you are putting us on danger as well". Health Manager, Shaheed Benazirabad

These stressors and uncertainties of being infected also affected provider-patient interaction during the initial phase of the outbreak and as it peaked. A service provider shared that:

"When a person is mentally unwell and is feeling uncertain, then s/he wouldn't even deliver what is normally expected of him/her. We were not able to take care of the patients then. We didn't

For peer review only - http://bmjopen.bmj.com/site/about/guidelines.xhtml

For peer review only

know whether the incoming patient is COVID positive or not. We just kept a distance and asked patient ailment. We had to save ourselves someway". Service Provider, Faisalabad

Fear of isolation was not reported by many HCWs. Only a few facility-based service providers reported to be stressed as they had to stay away from their family members, or by being alone.

"She [my co-worker] took her medicine regularly for 14 days at home. Then her [COVID19] test was conducted after 14 days and it came negative. But still she was very worried and tensed, and she was crying that she had to stay away from her kids". Service Provider, Khairpur

Mixed responses were reported by the LHWs in terms of stigma faced by them. According to some LHWs, community members resisted their entry into the households; whereas, senior or experienced LHWs reported fewer such issues in the field.

"During corona [peak] days, when we [I and my LHW] used to go out, people used to complain a lot. A huge pandemic has spread and you are coming to our house, so how do we know whether you have it [COVID19] or not; you can infect us. We felt this kind of resistance a lot in field even though we used to go with PPEs (sanitizers and masks)… at some places, sometimes it [people's reaction] was normal and some people gave very positive responses". Lady Health Supervisor, Rawalpindi

1.3 Challenges regarding patients:

A major challenge that was a source of stress and frustration among HCWs was uncooperative patients. Facility-based staff were concerned that patients were reluctant to wear face-mask or to adhere physical distancing within the premises of health facilities, and at times, they became offensive when they were insisted to take protective measures for COVID19.

"Our stressor was our patients because they were not ready to understand the situation. They didn't cooperate with us. They thought we didn't want to treat them. We get the announcement done here in our local areas and also pasted the posters. We also wrote on the board outside to 'wash your hands' and 'wear mask in hospital'; but we had to tackle a lot of troubles like these. Health Manager, Gujrat

Due to illiteracy, people did not believe in the existence of any viral outbreak, and they considered such protective measures as tactics of health workers to avoid service delivery.

"Only those women are worried who are a little bit educated but mostly aren't, 99% of people are don't have any fear and they don't believe in corona existence and they think that we are making them stupid". Health Manager, Mirpur Khas

Early on, another challenge faced by facility-based staff (both service providers and managers) was to conduct COVID19 test of suspected patients. According to them, people were extremely reluctant to share any details or willing to undergo COVID19 test. This was primarily due to the prevalent myths and misconceptions regarding COVID19 at the beginning of this outbreak.

"People used to bring sticks to beat us because it is a rural area and villagers used to say that our patients didn't have anything and they asked us to go away. We had to call assistant commissioner of that area than he used to send police. After that we were able to take samples from them. It was mental torture for us. Believe me, when we tell people that we are about to take

For peer review only - http://bmjopen.bmj.com/site/about/guidelines.xhtml

their samples, tell us your mobile number and Identification Card number etc. I swear they curse us and run away" Service Provider, Sargodha

1.4 Stress due to lack of availability of personal protective equipment

Lack of availability of personal protective equipment or supplies put both facility- and community-based HCWs under immense stress of being infected. Even facility-based HCWs who were seconded to a quarantine centre expressed concerns about unavailability of PPE.

"I had duty in quarantine for 3 months then I didn't have any kit or PPE, or mask or glove. I got a call and they told me to come on duty that day at 2 so I started crying because both my parents were home. We heard about doctors and XYZ deaths. I was scared and didn't have any protection kit". Service Provider, Sialkot

In the absence of PPEs, some of the facility-based service providers and lady health workers made use of the scarf or linen to cover themselves and their patients' face or a community member during household visit, respectively; while majority them reported to have it purchased on their own.

"Obviously, there weren't many masks available for us even and for patients. So any piece of cloth, or scarf, we use to make them cover their mouths and nose. Males sometimes used linen or face mask". Health Manager, Ghotki

1.5 Stress due to excessive work-load

Increased work-load was mainly the concern of field-based staff (LHWs and LHS) and for those who were given additional responsibilities like secondment to COVID19 isolation centre. Field teams had to locate suspected cases at a short notice. Due to lack of proper transportation, especially during lockdown, made things were quite stressful and exhaustive.

"Everybody was busy running to and fro because [COVID19] cases would come without notice. Doctor would get anxious and ask us to collect data and pictures [of patients]. We had to go to LHS and LHW. Lady Health Supervisor, Rawalpindi

On the contrary, the work load of facility-based service providers reduced considerably during the initial phase in the health facilities as people was reluctant to go to health facilities in the fear of COVID and certain prevalent myths.

"Yes, it (health facility) was working but patients were very less. Usually we have 200 or 300 patients but during corona we had only 30 or 40 patients. Even there was news circulating in news channels that poison injection were being given in the hospitals to corona patients. So people were really afraid and due to these kinds of rumours they stopped coming to hospitals". Service Provider, Sargodha

2. Available support mechanism to address mental health needs of health workforce

2.1 Awareness raising of healthcare workers

All facility- and community-based HCWs reported to have received awareness raising trainings that were organised by the department. The trainings primarily focused on preventive aspect of COVID19: characteristics of virus, signs and symptoms, modes of transmission, population at risk, preventive and protective measures, quarantine guidelines, and its testing.

For peer review only - http://bmjopen.bmj.com/site/about/guidelines.xhtml

"The purpose of training was to screen yourself, keep yourself safe, save the people of your organization, and also protect community; things that can control the disease. If a patient has symptoms, tell them where to do test and how it is done". Service Provider, Khairpur

However, participants from all three study groups unanimously informed that they had not received any training on mental health except for one lady health supervisor.

"In trainings, we talked on all issues, right. But, the main issue that was not touched is people's mental condition during corona. Nobody ever tried to assess that amongst us. We have never ever tried to understand people mental level or their mental condition or our feelings". Lady Health Supervisor, Rawalpindi.

According to all HCWs, increased awareness and declining COVID19 cases over time mitigated the fear and stress among healthcare workers.

"Right now [health] workers are not depressed they are normal and doing their routine work. They were depressed and worried in the beginning but now they are normal due to awareness campaigns". Health manager, Shaheed Benazirabad

2.2 Provision of personal protective equipment

The PPEs were provided to the health workers with some delays, after few weeks of the outbreak in the country. The use of these PPEs, combined with other protective measures of COVID19, played a major role in reducing fear and stress among healthcare workers.

"We displayed the posters outside of hospitals - informing people not to come to the hospital without any reason. Maintain social distancing, wash hands, make soaps available everywhere and made hand sanitizers available. That's how we coped up with the stress. To be very frank, in the beginning everything was very difficult for me and for them [health workers]". Service Provider, Multan

"Firstly, protection, masks, shields etc. there was a shortage before but now they are available. There is also awareness now, we are seeing that there are fewer patients in Pakistan compared to other countries. All these things are effective for mental health". Service Provider, Sialkot

2.3 Social support from co-workers and family members

Provision of social support by co-workers - including colleagues and supervisors – emerged as a common response mechanism to mitigate the fear, stress and anxiety among both facility- and community-based HCWs. During the early phase of this outbreak when stress, anxiety and fear was high, staff members used to discuss and provide social support to each other. Senior HCWs often used to enquire about challenges being faced by their colleagues, including if they are emotionally disturbed. They used to console them to help overcome any kind of fear, stress or anxiety pertaining to COVID19.

"We just provided them emotional support. I educated staff members that we are health care providers, how could we help people if all of us become a victim of mental health issues. We have to be strong because we are on frontline. We don't have to be fearful. Along with that we used to appreciate workers. What if they themselves become depressed then who would work. I told them that corona has no limit so we will have to live with it". Health Manager, Khairpur

For peer review only - http://bmjopen.bmj.com/site/about/guidelines.xhtml

A couple of HCWs acknowledged that they used to consult and take frequent advice from ex-physiatrist mates to overcome stress, and some received support from their family members.

"I was in contact with a psychiatrist so in that way I used to get constant help from there. I personally took many sessions". Service Provider, Faisalabad

"My husband kept on telling me that the night that is in the grave is not outside, so stop thinking about it and the major support that my husband gave me was it is a death by martyrdom so I used to get relaxed from that as well". Lady Health Supervisor, Rawalpindi

The emotional support was provided in several ways. Some of the consoling approaches were: a) reinforcement of the use of PPEs and COVID19 SOPs; b) reiterated the purpose of being a health worker; c) ensured frequent communication with the stressed person and giving assurance of being with them; d) motivated colleagues by giving their own example; e) encouraging others to keep themselves busy if they are stressed. Importantly, some used religious healing perspective by linking everything with God 'Allah' that He will take care of everything.

"All you have to do is care, and this is a type of help and if any patient gets well with our help it would be a blessing from Allah, living and dying is Allah's decision, we can only take care so take care and help others, you will be rewarded InshaAllah [if Allah wills] so we say all this to each other and we give support to each other". Service Provider, Dadu

"I was continuously in contact with her [COVID19 positive health worker] and tell her to take care of herself and she was telling her children whether test will come negative or not. She was scared that she had corona and she would die. Yes, we told her God will help you. We have doctors with us and our district manager and medical superintendent are with us, they were talking to her continuously. They were also consoling here that corona will end why you are worried?" Service Provider, Khairpur

"Reassuring, giving trust, and with positive thinking for the future, telling them that we are messengers, Allah has placed us to serve these people, to take care of them, to tell them preventions". Lady Health Supervisor, Rawalpindi

A few facility-based HCWs kept themselves busy in extra-curricular activity to deal with their stress like reading or even social media.

"I kept myself engaged because whenever I was free then I would get a lot stress when you hear news etc. I was studying and spent most of my time with my co-worker. And whenever I would go home, I would keep myself engaged mentally". Service Provider, Sialkot

2.4 Managerial support from supervisors

According to a majority of service providers and lady health workers, their supervisors/managers provided a lot of support in managing work-related issues that were causing stress. The supervisors primarily managed issues of their teams by themselves, or at times, by engaging a higher (district) administrative health authorities. Effective communication with team that included constant motivation and awareness raising was instrumental in addressing day-to-day challenges. At some instances, health managers also offered intermittent breaks to the health workers who were unwell or became exhausted due to work. A couple of respondents also

For peer review only - http://bmjopen.bmj.com/site/about/guidelines.xhtml

mentioned that the health facility was closed for a few days when the COVID19 positive staff was quarantined.

"We fulfil their demands, if there is a salary issue, if they haven't received their salary from 2 to 3 months, we write it to DHO that they will not do work with interest so they should be given their salary on time and they forward it to upper department and their problems are solved. Service Provider, Dadu

"I counsel them that see this is difficult for us; you cooperate with me and I will cooperate with you. If you have any problem then you can tell me and take off. If there is no issue then take half day off but come to work next day. So I tried to make them relax like this". Health Manager, Karachi

3. Mental health needs of healthcare workers and recommended solutions

3.1 Safe and conducive work environment

Safe working environment was identified as one of the prominent needs of both facility-based and community-based HCWs. It primarily meant timely provision of PPE kits that should include gloves, face-masks, and hand sanitizers, and provision of security especially to deal with aggressive patients.

"We should have received PPE kits in the beginning before [COVID19] peak time. When cases were increasing in China, since then we should have made ourselves prepared for this". Service Provider, Multan

"Security should be given especially in this COVID situation. Because when patients come to the health facility and the doctor fails to examine them then they create a scene or become aggressive, and misbehave". Service Provider, Sialkot

Primarily, facility-based HCWs who were assigned additional responsibilities related to COVID19 expressed a dire need of intermittent breaks under certain circumstances – for example, when a worker is ill, exhausted due to increased work-load, or experiences any kind of stress.

"If someone appears COVID-19 positive then that person is placed in 14 days quarantine. So if he is already in a stressful condition, his mental health isn't able to hold so at least until he is normalized, there should be some relief given". Service Provider, Faisalabad

"They [health workers] say that how can we perform our normal duties and COVID ward both simultaneously? So they should be given holidays. Our demand is that our staff should be given more holidays and good packages and some financial bonuses should be provided". Health Manager, MirpurKhas

3.2 Awareness about evolving situation of COVID19 and corrective actions

A majority of facility- and community-based HCWs expressed the need for having updated information about the evolving situation regarding COVID19. They also identified the need of receiving timely guidelines about the corrective actions to deal with constantly changing situation.

For peer review only - http://bmjopen.bmj.com/site/about/guidelines.xhtml

“There should have been sessions about the forthcoming epidemic. Like this what is this disease, what are its consequences, how to deal with it. There should’ve been such learnings that they prepare their workers. They just said that ‘wear masks’, and what’s being told on TV”. Mealth Manager, Khairpur

3.3 Psychosocial support from specialized mental healthcare workers:

Even though social support emerged as a key response-measure used by HCWs to cope with the prevailing stress, getting psychosocial help from specialized mental health professional was still identified as a need and future recommendation by majority of the study participants. Various suggestions were given to have such support in-place for the HCWs, which included: a) having a full-time specialist based district or sub-district level to provide needs-based support, b) conduction of periodic sessions on mental health, c) mobile team of psychologist to provide periodic support of health staff; and d) making mental health component part of the COVID19 trainings.

“Mental health is a drawback here. There should be a proper person, who should, first of all, educate the staff, that how will you improve the mental health of the people. Then, some training should be arranged there, meaning, there should be a proper person sitting there, right, like some psychiatrist, or some people like these”. Lady Health Supervisor, Rawalpindi

“In my opinion, CEO of our hospital should depute someone from DHQ like 3 sessions in a week so that they can give sessions to healthcare workers, perform counselling and try to know their inner problems due to which they are disturbed so that their problems can be solved. And provide them relief if it is related to salary or any other issue”. Service Provider, Sargodha

Some facility-based HCWs suggest use of mobile application to provide information about mental health and well-being.

“They should conduct seminars and this is the era of multimedia everyone has mobile and there should be more content on multimedia and social media about mental health so that we become mentally strong and cater things properly. There should be seminars and sessions and government should launch an app regarding this so that we get information from it regarding mental and physical health and keep on updating it”. Health Manager, Gujrat

On the other hand, facility-based healthcare workers expressed the need of capacity building to provide adequate psychosocial support to co-workers and to their patients, as needed. These trainings are also expected to help them guide general patients or community people to follow COVID19 SOPs.

“No, patients aren’t believing, that’s why psychiatrist is important and there should be training provided by the psychiatrist. Our staff should be provided psychological training, we should know what they [patients] are saying and what their opinions are so then we can handle them accordingly”. Health Manager, MirpurKhas

3.4 Appreciation and recognition of their work in the pandemic crisis:

Both facility- and community-based HCWs demanded recognition and appreciation for the additional work that they had to do during COVID19 crisis. This need was particularly highlighted by those who were given additional responsibilities like secondment at isolation centres, contact

For peer review only - http://bmjopen.bmj.com/site/about/guidelines.xhtml

tracing, and COVID19 testing of suspected cases. This recognition was demanded in the form of monetary incentive for risking their lives, or a letter of appreciation by the health department, or a continuous encouragement from their administrative leadership.

"Lady Health Workers are already doing extra work, putting their lives in danger, should have gotten some extra benefits for this, even if they had gotten any certificate. Even if we get one praise, they want to sacrifice everything". Lady Health Supervisor, Rawalpindi

"I would say that government should facilitate the doctor, increase the allowance, give them a complete personal kit, if someone dies, government should support their family, so this should be on government level, so doctors also work motivated… I would want that government should give full support, financially and morally". Service Provider, Khairpur

Discussion:
The current body of existing literature revolved predominantly around the mental health and well-being of HCWs who are involved in provision of specialised COVID-related health services. It largely neglects the PHCWs who are the first point of contact for patients and are also involved in wide range of COVID19-related activities, which in turn can impact their mental health. Our study is unique that it explored the mental health impact of COVID19, health system response, and sought suggestions and recommendations from the PHCWs to address the mental health needs of PHCWs in pandemic crisis.

Reportedly, constant fear, anxiety and stress of being infected and infecting others was immense at onset of COVID19 outbreak and during its peak time. This was primarily triggered by the lack of information about the virus and its management, false rumours regarding COVID19, media-hype, lack of availability of PPEs, and not being able to distinguish between COVID19 positive and negative patients. Evidence show that exaggeration by media tend to negatively affect the mental health of HCWs [45,46]. Similarly, lack of knowledge about COVID19 infection is likely to increase anxiety/depression among physicians[47] and inadequate precautionary measures in the workplace causes fear and anxiety among HCWs[48].

Another important concern causing distress and frustration among healthcare workers was non-compliance of COVID19 SOPs by incoming patients and their companions. According to service providers, low literacy in rural populations, lack of awareness and non-serious attitude towards COVID19 were the primary reasons for this non-adherence. This is consistent with another study from Pakistan that identified non-compliance of COVID19 SOPs as one of the reasons for stress and anxiety among HCWs[34]. Misconceptions and stigma attached to COVID19 also made it immensely challenging for HCWs for contact tracing and COVID19 testing on suspected patients. In extreme cases, people became aggressive and reacted violently to HCWs. Similar incidents have been reported in Pakistan and other countries [49,50]. Such incidents have been associated with work-related stress, burn out, and post-traumatic stress disorder, which in turn negatively affect job performance and quality of services [51].

The mental health impact of COVID19 tapered off over time among PHCWs. PHCWs attributed the reduction of stress and anxiety the support provided by the government in the form of awareness raising sessions regarding COVID19 and its preventive measures, and provision of PPE. More importantly, social support by co-workers (including supervisors) emerged a key factor that alleviated the level of stress and fear among healthcare workers at the beginning when it was very high. Social support by co-workers has also been reported as an effective response-mechanism to deal with prevalent mental health issues among HCWs COVID19 crisis [52,53]. Given

For peer review only - http://bmjopen.bmj.com/site/about/guidelines.xhtml

its significance, fostering a systematic mechanism of social support within healthcare system has also been recommended to safeguard mental health of HCWs during the pandemic[53]. Consistent with other studies [9], patient visits at PHCWs significantly reduced due to the fear of COVID19 infections among general population. Work-load was only a concern of PHCWs were assigned additional task regarding COVID19, and these HCWs reported experiences of exhaustion due to additional responsibilities. They also demanded recognition and appreciation in the form official appreciation letter or monetary incentives for risking their lives. This finding is consistent with other studies where HCWs expressed similar needs [18,49].

In the end, PHCWs rendered few recommendations to address their mental health needs in pandemic crisis. Firstly, they expressed the necessity of having a safe and conducive working environment that included timely provision of PPE for all staff members, safety and security of HCWs to deal with difficult patients, and intermittent breaks – especially for those who are assigned additional COVID19-related responsibilities. The positive effect of safe and conductive work environment on mental health is already discussed above. Second recommendation was around preparedness of HCWs through continuous awareness raising and corrective actions about the evolving pandemic situation. Increased knowledge is also linked with reduced level of stress and anxiety among healthcare workers[47]. Thirdly, they highlighted the need for psychological counselling from mental health professionals and suggested different approach to fulfil this need. Call centres have been established in many countries – including Pakistan[54] - to provide needs-based psychosocial support to HCWs. In Pakistan, this programme perhaps need to expand their scope and provide more targeted services to the PHCWs. Additionally, HCWs requested the need for training to be able to provide psychosocial support to co-worker and dealing with difficult patients or people in the communities. There has already been a debate about potential role of community health workers in the provision of psychosocial support to people during pandemic crisis [55]. The existing "WeCare" programme of the Pakistani government could also be effectively integrated with PHC settings to assist HCWs in providing support to the incoming patients. Lastly, a key need identified by the HCWs, especially those who were assigned additional tasks related to COVID19, was appreciation and recognition for their work in pandemic crisis. They particularly stressed on developing a formal mechanism for the acknowledgement of their efforts via appreciation letter or in the form of monetary incentive or risk allowances. Similar need was also identified in other studies as well [56].

Strengths and Limitations:

In terms of strengthens, our study used gather experiences of a neglected group HCWs that belonged to PHC settings. It uses an inductive interpretative approach to achieve study objectives by gathering information from diverse group of service providers and health managers, including lady health workers. Importantly, this research revealed the perspective and experience of HCWs from the rural settings which are largely neglected in the current body of knowledge. It allowed researchers to look for converging and diverging lines of inquiry to identify common themes/ concepts and incongruence between data sources. On the contrary, a major limitation was that all interviews were conducted over phone which may have constrained rapport building with participant and obtain no-verbal cues during the interviews. In addition, the interviewees might have been distracted during interviews by the activities in their environment and might have experienced fatigue due to long telephonic interviews. Another limitation was that the study was conducted with a focus on public sector hospitals in only two provinces of Pakistan (Sindh and Punjab), which would limit the transferability of the findings to other provinces[57]. However, this study may provide insights for similar PHC settings across Pakistan that are interested in understanding the mental health impact and mental health needs of health service providers during Covid-19. Lastly, the study was unable to carry out member checking with study participants, which may have affected the validity of study findings.

For peer review only - http://bmjopen.bmj.com/site/about/guidelines.xhtml

Conclusion:
Our study concludes that the stress and anxiety among primary healthcare workers was immense at the beginning of this outbreak or when the COVID19 transmission was at its peak. However, it tapered-off as they become more aware of the virus, receive PPEs, and number of COVID19 cases decreased in their community. Mental health of either health worker or patients were not covered in the trainings offered to them – neither had they had access to any professional psychosocial support. Our study identified a need for a comprehensive support that should be provided to PHCWs in a timely manner, including informational support (e.g. continuous awareness about evolving situation and corrective actions); safe and conducive work environment (e.g. provision of personal protective equipment, personal security to deal with violent reaction of community people due to stigma); organisational support (e.g. intermittent breaks, appreciation and recognition in the form of appreciation letters or monetary incentives (especially for those who are assigned additional responsibilities); and frequent professional psychosocial support to cope with stress but also capacity building trainings on mental health that will enable them deal with difficult patients or community people.

Contributions:
WH and BIA conceptualised the study with inputs from BK, ZF and SS. WH, BIA, BK, ZF, and ASF developed interview guide. HJ and MW facilitated in conducting the study. WH and ASF performed data analysis. BK and ZF reviewed the themes and sub-themes again. WH took the lead in writing the manuscript. All authors critically reviewed the manuscript and provided feedback in shaping the write-up, analysis and presentation of results.

Conflicts of interest: None

Funding: This work was co-funded by the Aga Khan University, Pakistan grant number 20051 and the World Health Organization grant number 202568710-1

Data sharing: The data will be available upon request

Ethics: Ethics approval was obtained from Aga Khan University (Ref. number: 2020-5186-11602) and National Bioethics Committee of Pakistan (Ref. Number: 4-87/COVID-45/NBC/20/393).

Acknowledgements: We are grateful to our implementing partners: Department of Health, Government of Sindh; Primary and Secondary Healthcare Department (P&SHD) and Specialized Healthcare & Medical Education Department from Government of Punjab for extending their full their support and guidance to conduct this survey. We are also thankful to all our study participants for sparing their precious time and made themselves available for the interviews. Last but not the least, we thank our data collectors and project management team: Zafar Dehraj, Imran Sheikh, Sajid Brohi, Noreen Afzal and Ghani Muhammad who managed the implementation of this study in a proficient manner during the pandemic.

For peer review only - http://bmjopen.bmj.com/site/about/guidelines.xhtml

Disclaimer:

The views expressed in this manuscript are those of the authors and contributors, and do not necessarily reflect those of the World Health Organization, or the organization to which the authors are affiliated.

For peer review only

For peer review only - http://bmjopen.bmj.com/site/about/guidelines.xhtml

Reference List

1. Li Q, Guan X, Wu P*, et al.* Early Transmission Dynamics in Wuhan, China, of Novel CoronavirusΓÇôInfected Pneumonia. *New England Journal of Medicine* 2020; 382:1199-1207.https://doi.org/10.1056/NEJMoa2001316

2. Center for Systems Science and Engineering. Coronavirus Resource Center. 27-4-2020. Baltimore, United States, Johns Hopkins University. 27-4-2020.

Ref Type: Online Source

3. World Health Organization. Health and Care Worker Deaths during COVID-19. 20-10-2021. Geneva, World Health Organization. 20-9-2021.

Ref Type: Online Source

4. Wu P, Fang Y, Guan Z*, et al.* The Psychological Impact of the SARS Epidemic on Hospital Employees in China: Exposure, Risk Perception, and Altruistic Acceptance of Risk. *Can J Psychiatry* 2009; 54:302-311.https://doi.org/10.1177/070674370905400504

5. Li Y, Scherer N, Felix L*, et al.* Prevalence of depression, anxiety and post-traumatic stress disorder in health care workers during the COVID-19 pandemic: A systematic review and meta-analysis. *PLoS ONE* 2021; 16:e0246454.https://doi.org/10.1371/journal.pone.0246454

6. Moitra M, Rahman M, Collins PY*, et al.* Mental Health Consequences for Healthcare Workers During the COVID-19 Pandemic: A Scoping Review to Draw Lessons for LMICs. *Frontiers in Psychiatry* 2021; 12:22.https://www.frontiersin.org/article/10.3389/fpsyt.2021.602614

7. Vizheh M, Qorbani M, Arzaghi SM*, et al.* The mental health of healthcare workers in the COVID-19 pandemic: A systematic review. *Journal of Diabetes & Metabolic Disorders* 2020; 19:1967-1978.https://dx.doi.org/10.1007%2Fs40200-020-00643-9

8. Dunlop C, Howe A, Li D*, et al.* The coronavirus outbreak: the central role of primary care in emergency preparedness and response. *BJGP Open* 2020; 4:bjgpopen20X101041.https://pubmed.ncbi.nlm.nih.gov/31992543

9. Rawaf S, Allen LN, Stigler FL*, et al.* Lessons on the COVID-19 pandemic, for and by primary care professionals worldwide. *European Journal of General Practice* 2020; 26:129-133.https://doi.org/10.1080/13814788.2020.1820479

10. Sarti TD, Lazarini WS, Fontenelle LF*, et al.* Qual o papel da Aten+º+úo Prim+íria +á Sa+¦de diante da pandemia provocada pela COVID-19? *Epidemiologia e Serviços de Saúde* 2020; 29.https://doi.org/10.5123/s1679-49742020000200024

11. Bhaumik S, Moola S, Tyagi J*, et al.* Community health workers for pandemic response: a rapid evidence synthesis. *BMJ Global Health* 2020; 5:e002769.http://gh.bmj.com/content/5/6/e002769.abstract

For peer review only - http://bmjopen.bmj.com/site/about/guidelines.xhtml

12. Gray DM, II, Joseph JJ, Olayiwola JN. Strategies for Digital Care of Vulnerable Patients in a COVID-19 World–Keeping in Touch. *JAMA Health Forum* 2020; 1:e200734.https://doi.org/10.1001/jamahealthforum.2020.0734

13. First in, last out - The role of family doctors in the fight against novel coronavirus. 2020. World Organization of Family Doctors, WONCA. 7-10-2021.
Ref Type: Online Source

14. Mash B. Primary care management of the coronavirus (COVID-19). *S Afr Fam Pract (2004)* 2020; 62:e1-e4.https://pubmed.ncbi.nlm.nih.gov/32242438

15. Waters R. Community Workers Lend Human Connection To COVID-19 Response. *Health Aff (Millwood)* 2020; 39:1112-1117.https://doi.org/10.1377/hlthaff.2020.00836

16. Donnelly C, Ashcroft R, Bobbette N*, et al.* Interprofessional primary care during COVID-19: a survey of the provider perspective. *BMC Family Practice* 2021; 22.https://doi.org/10.1186%2Fs12875-020-01366-9

17. Ashcroft R, Sur D, Greenblatt A*, et al.* The Impact of the COVID-19 Pandemic on Social Workers at the Frontline: A Survey of Canadian Social Workers. *The British Journal of Social Work* 2021; 52:1724-1746.https://doi.org/10.1093/bjsw/bcab158

18. Strengthening the frontline: How primary health care helpshealth systems adapt during the COVID-19 pandemic. 10-2-2021. OECD Policy Responses to Coronavirus (COVID-19). 12-5-2021.
Ref Type: Report

19. National Center for Immunization and Respiratory Diseases (NCIRD) Division of Viral Diseases. Engaging Community Health Workers to Support Home-based Care for People with COVID-19 in Low-Resource Non-U.S. Settings. 7-4-2021. United States, Center for Disease Control and Prevention. 12-5-2021.
Ref Type: Online Source

20. De Kock JH, Latham HA, Leslie SJ*, et al.* A rapid review of the impact of COVID-19 on the mental health of healthcare workers: implications for supporting psychological well-being. *BMC Public Health* 2021; 21:104.https://doi.org/10.1186/s12889-020-10070-3

21. Tremblay S, Castiglione S, Audet LA*, et al.* Conducting Qualitative Research to Respond to COVID-19 Challenges: Reflections for the Present and Beyond. *International Journal of Qualitative Methods* 2021; 20:16094069211009679.https://doi.org/10.1177/16094069211009679

22. Zace D, Hoxhaj I, Orfino A*, et al.* Interventions to address mental health issues in healthcare workers during infectious disease outbreaks: A systematic review. *J Psychiatr Res* 2021; 136:319-333.https://www.sciencedirect.com/science/article/pii/S0022395621000868

23. Torjesen I. Covid-19 will become endemic but with decreased potency over time, scientists believe. *BMJ* 2021; 372:n494.http://www.bmj.com/content/372/bmj.n494.abstract

For peer review only - http://bmjopen.bmj.com/site/about/guidelines.xhtml

24. Phillips N. The coronavirus is here to stay — here's what that means. *Nature* 2021; 590:382-384.https://doi.org/10.1038/d41586-021-00396-2

25. Gunnell D, Appleby L, Arensman E, *et al.* Suicide risk and prevention during the COVID-19 pandemic. *The Lancet Psychiatry* 2020.https://doi.org/10.1016/S2215-0366(20)30171-1

26. Population Reference Bureau. 2021 World Population Data Sheet. 2021. Washington DC, Population Reference Bureau. 28-9-2021.
Ref Type: Online Source

27. National Information Technology Board. COVID-19 Dashboard. 27-4-2020. Islamabad, Government of Pakistan. 27-4-2020.
Ref Type: Online Source

28. Hassan A, Mahmood K, Bukhsh HA. Healthcare System Of Pakistan. *International Journal of Advanced Research and Publication* 2017; 1:170-173.https://www.ijarp.org/published-research-papers/oct2017/Healthcare-System-Of-Pakistan.pdf

29. Waris A, Atta UK, Ali M, *et al.* COVID-19 outbreak: current scenario of Pakistan. *New Microbes and New Infections* 2020; 35:100681.http://www.sciencedirect.com/science/article/pii/S2052297520300330

30. Khalid A, Ali S. COVID-19 and its Challenges for the Healthcare System in Pakistan. *Asian Bioethics Review* 2020; 12:551-564.https://dx.doi.org/10.1007%2Fs41649-020-00139-x

31. Fullman N, Yearwood J, Abay SM, *et al.* Measuring performance on the Healthcare Access and Quality Index for 195 countries and territories and selected subnational locations: a systematic analysis from the Global Burden of Disease Study 2016. *Lancet* 2018; 391:2236-2271.https://doi.org/10.1016/S0140-6736(18)30994-2

32. Abdullah MA, Mukhtar F, Wazir S, *et al.* The Health Workforce Crisis in Pakistan: A Critical Review and the Way Forward. *World Health & Population* 2014; 15:4-12.https://www.longwoods.com/product/24031

33. Asim SS, Ghani S, Ahmed M, *et al.* Assessing Mental Health of Women Living in Karachi During the Covid-19 Pandemic. *Frontiers in Global Women's Health* 2021; 1:594970.https://doi.org/10.3389/fgwh.2020.594970

34. Sandesh R, Shahid W, Dev K, *et al.* Impact of COVID-19 on the Mental Health of Healthcare Professionals in Pakistan. *Cureus* 2020; 12:e8974.https://pubmed.ncbi.nlm.nih.gov/32775056

35. Arshad MS, Hussain I, Nafees M, *et al.* Assessing the Impact of COVID-19 on the Mental Health of Healthcare Workers in Three Metropolitan Cities of Pakistan. *Psychol Res Behav Manag* 2020; 13:1047-1055.https://pubmed.ncbi.nlm.nih.gov/33244279

36. Government of Punjab. National Programme for Family Planning & Primary Health Care, Pakistan. 2011. National Programme for Family Planning & Primary Health Care. 4-5-2015.
Ref Type: Online Source

For peer review only - http://bmjopen.bmj.com/site/about/guidelines.xhtml

37. Perry H, Zulliger R, Scott K, Javadi D, Gergen J, Shelley K et al. Case Studies of Large-Scale Community Health Worker Programs: Examples from Afghanistan, Bangladesh, Brazil, Ethiopia, Niger, India, Indonesia, Iran, Nepal, Pakistan, Rwanda, Zambia, and Zimbabwe. 2017. Baltimore, United States, Maternal and Child Health Integrated Program. 1-9-2022.
Ref Type: Report

38. Creswell JW, Poth CN. Qualitative Inquiry and Research Design: Choosing Among Five Approaches. Fourth Edition ed. SAGE Publishing; 2017.

39. Yin RK. Case Study Research and Applications: Design and Methods. Sixth Edition ed. SAGE Publishing; 2017.

40. National Information Technology Board. COVID-19 Dashboard. 30-8-2022. Islamabad, Government of Pakistan. 30-8-2022.
Ref Type: Online Source

41. Fusch PI, Ness LR. Are We There Yet? Data Satur et? Data Saturation in Qualitative Research. *The Qualitative Report* 2015; 20:1408-1416.https://nsuworks.nova.edu/cgi/viewcontent.cgi?article=2281&context=tqr

42. NVivo qualitative data analysis software [QSR International Pty Ltd.; 2015.

43. Hsieh HF, Shannon SE. Three Approaches to Qualitative Content Analysis. *Qual Health Res* 2005; 15:1277-1288.https://doi.org/10.1177/1049732305276687

44. Tong A, Sainsbury P, Craig J. Consolidated criteria for reporting qualitative research (COREQ): a 32-item checklist for interviews and focus groups. *Int J Qual Health Care* 2007; 19:349-357.https://doi.org/10.1093/intqhc/mzm042

45. Su Z, McDonnell D, Wen J*, et al.* Mental health consequences of COVID-19 media coverage: the need for effective crisis communication practices. *Globalization and Health* 2021; 17:4.https://doi.org/10.1186/s12992-020-00654-4

46. World Health Organization. Health workforce policy and management in the context of the COVID-19 pandemic response: interim guidance, 3 December 2020. 3-12-2020. Geneva, World Health Organization. 10-10-2021.
Ref Type: Report

47. Amin F, Sharif S, Saeed R*, et al.* COVID-19 pandemic- knowledge, perception, anxiety and depression among frontline doctors of Pakistan. *BMC Psychiatry* 2020; 20:459.https://doi.org/10.1186/s12888-020-02864-x

48. Hoernke K, Djellouli N, Andrews L*, et al.* Frontline healthcare workers' experiences with personal protective equipment during the COVID-19 pandemic in the UK: a rapid qualitative appraisal. *BMJ Open* 2021; 11:e046199.http://bmjopen.bmj.com/content/11/1/e046199.abstract

49. Martins RS, Bhatti OA, Mian AI. Violence Against Health Care Workers in Pakistan During the COVID-19 Pandemic. *JAMA Health Forum* 2020; 1:e201263.https://doi.org/10.1001/jamahealthforum.2020.1263

For peer review only - http://bmjopen.bmj.com/site/about/guidelines.xhtml

50. Bhatti OA, Rauf H, Aziz N*, et al.* Violence against Healthcare Workers during the COVID-19 Pandemic: A Review of Incidents from a Lower-Middle-Income Country. *Ann Glob Health* 2021; 87:41.https://pubmed.ncbi.nlm.nih.gov/33977084

51. Shaikh S, Baig LA, Hashmi I*, et al.* The magnitude and determinants of violence against healthcare workers in Pakistan. *BMJ Global Health* 2020; 5:e002112.http://gh.bmj.com/content/5/4/e002112.abstract

52. Al-Mansour K, Alfuzan A, Alsarheed D*, et al.* Work-Related Challenges among Primary Health Centers Workers during COVID-19 in Saudi Arabia. *Int J Environ Res Public Health* 2021; 18:1898.https://pubmed.ncbi.nlm.nih.gov/33669440

53. Labrague LJ. Psychological resilience, coping behaviours and social support among health care workers during the COVID-19 pandemic: A systematic review of quantitative studies. *J Nurs Manag* 2021; n/a.https://doi.org/10.1111/jonm.13336

54. Ministry of National Health Services Regulation and Coordination (MoNHSRC). We CARE. 2020. Islamabad, Government of Pakistan. 18-9-2021.

Ref Type: Online Source

55. Mistry SK, Harris-Roxas B, Yadav UN*, et al.* Community Health Workers Can Provide Psychosocial Support to the People During COVID-19 and Beyond in Low- and Middle- Income Countries. *Frontiers in Public Health* 2021; 9:800.https://www.frontiersin.org/article/10.3389/fpubh.2021.666753

56. Shaukat N, Ali DM, Razzak J. Physical and mental health impacts of COVID-19 on healthcare workers: a scoping review. *Int J Emerg Med* 2020; 13:40.https://pubmed.ncbi.nlm.nih.gov/32689925

57. Donmoyer R. Generalizability and the Single-Case Study. In: Gomm R, Hammersley M, Foster P, editors. Case Study Method. SAGE publications Ltd.; 2009.

For peer review only

For peer review only - http://bmjopen.bmj.com/site/about/guidelines.xhtml

COVID-19: psychosocial and mental health needs of health workers during the outbreak management in Pakistan

IN-DEPTH INTERVIEW GUIDE FOR HEALTH MANAGERS AND SERVICE PROVIDERS

SECTION 1: PARTICIPANT CONTACT SHEET			
**S.no**	**Questions**	**Responses**	**Skip**
Please complete before the interview.			
101	Name of province	Sindh 1<br>Punjab 2	
102	Name of district		
103	Name of TALUKA / Tehsil		
104	Name of Union Council		
105	Type of health facility	Basic Health Unit (BHU) 1<br>Rural Health Centre (RHC) 2<br>Taluka Head Quarter (THQ) 3<br>District Head Quarter (DHQ) 4<br>Tertiary care hospital 5<br>Specialised Isolation Centre 6	
106	Name of health facility / Hospital		
107	Date of interview	______ / _______ / _____________<br>dd mm yyyy	
**SECTION 2: PARTICIPANTS PROFILE**			
201	Sex	Male 1<br>Female 2	
202	Marital status	Never married 1<br>Currently married 2<br>Divorced 3<br>Separated 4<br>Widowed 5	
203	Age (in years)	Years [ ][ ]	
204	Number of living children	Children [ ][ ]	
205	Do you live in a nuclear family or joint family?	Nuclear 1<br>Joint 2	
206	Highest level of education completed?	Matriculation 1<br>Intermediate 2<br>Bachelors 3<br>Masters / M.Phil. 4<br>PhD 5	
207	Professional qualification	Lady Health Visitor 1<br>Lady Health Worker 2	

Version 1.0 Last updated 29th June 2020

For peer review only

		Midwife	3	
		Nurse	4	
		M.B.B.S	5	
208	What is the current position you hold in the organization?	Lady health supervisors (LHSs)	1	
		Managers from secondary level health facility (DHQ/THQ)	2	
		Medical superintendent of tertiary care teaching hospital	3	
		Specialist from Field isolation center, Karachi	4	
		Others specify_____________________	5	
209	How long you have been working in this health facility / catchment area?	Years [ ][ ]		
210	What is the total years of your professional experience?	Years [ ][ ]		

For peer review only

For peer review only - http://bmjopen.bmj.com/site/about/guidelines.xhtml

No.	Questions	Responses	Probe
**1**	**THEME : Understanding psychological impact of covid-19 on healthcare workers**		
	**First, I would like to ask you some questions to understand the psychological impact of covid-19 on healthcare workers**<br>**The psychological adverse outcomes may include stress, anxiety, depression, burnout, post-traumatic stress disorder, fear-triggered panic attacks, psychosomatic symptoms, psychological violence, fatigue, stigma etc.**		
1.1	How healthcare workers feel about working under tremendous pressure with COVID -19 situation?		Probes:<br>• Can you use example to explain the situation?
1.2	In your opinion, how has COVID-19 affected mental health of healthcare workers?		Probes:<br>• How healthcare workers are dealing with anxiety and fear related to managing COVID-19 inpatients?<br>• Is it affecting healthcare workers both negatively and positively?
1.3	Can you specify the reasons for adverse psychological outcomes among healthcare workers?		Probes:<br>• Reasons related to procedural changes (increased workload, long working hours)
1.4	How negative emotions among healthcare workers will affect the overall functioning of organisations/ institutions?		Probes:<br>• Do you think negative emotions among health worker may lead to poor health worker attention, understanding and decision-making ability
1.5	Do you think health workers have capacity to cope with psychological risk?		Probes:<br>• If yes, what are the coping mechanisms of healthcare workers?<br>• If no, why do you think healthcare workers lack capacity to cope with psychological risk?
1.6	Do you think psychological needs of population has changed in recent times?		Probes:<br>• How health system is responding to those psychological needs?<br>• Are health workers prepared to address changing psychological needs of patients with covid-19?
**2**	**THEME: Identifying psychological needs of health workforce involved in covid-19 crisis**		
	**Now I would like to ask some questions to understand the psychological needs of health workforce involved in covid-19 crisis. The psychological needs of healthcare workers may include enabling working environment, safe and secured hospital environment, adequate staffing, support from mentors and floor managers, support from mental health counsellors, etc.**		
2.1	What are the psychological needs of health workforce involved in covid-19 crisis		Probes:<br>• How did you get to know about their psychological needs?

For peer review only

For peer review only - http://bmjopen.bmj.com/site/about/guidelines.xhtml

2.2	Do you think healthcare workers are able to talk about their psychological needs and concerns?		Probes:<br>• If yes, please share examples of some instances where healthcare workers have shared their psychological needs<br>• If no, why healthcare workers are not able to talk about their concerns?
2.3	As a ______, how do you see your role in identifying the psychological needs of the health workforce		
**3**	**THEME: Support mechanisms available to address mental health needs of healthcare workers**		
	I would like to know the support mechanisms/ processes available to address mental health needs of healthcare workers. These support mechanisms may include providing mental health support services, delivering counselling sessions, supporting workers through mental health helpline, etc.		
3.1	What are the mechanisms available to support the mental health needs of healthcare workers?		Probes:<br>• What interventions are currently used to support the mental health needs of healthcare workers?<br>•
3.2	Do you think healthcare workers are able to receive adequate mental health support with these processes?		Probes:<br>• Are they effective? Are these interventions appreciated by current health workforce?
3.3	Do you think your institution/health facility is prepared to manage mental health needs of the healthcare workers?		Probes:<br>• If yes, please share a recent experience whereby mental health needs of a health worker were adequately addressed.<br>• Do you think health system is currently prepared to address the mental health needs of health care workers<br>○ If yes, why? If no, why?
3.4	Have you received any formal training/ session on supporting mental health needs of the healthcare workers?		Probes:<br>• How many times the training was conducted and for whom?<br>• Can you tell me the objective of each training?<br>• What aspects were covered in the training?<br>• Who conducted those trainings?<br>• What was the duration of each training?
3.5	As a ______, how do you see your role in addressing the psychological needs of the health workforce		Probes:<br>• How do you provide the support to your staff?<br>• Do you feel confident while addressing the mental health needs of your staff?

For peer review only - http://bmjopen.bmj.com/site/about/guidelines.xhtml

			• How do you feel about your capacity in terms of addressing psychological needs of your staff<br>• How effective it is?
4	**Theme: Understanding challenges in addressing mental health needs of healthcare workers**		
	Now I would like to understand the challenges you face while addressing mental health needs of health care workers like lack of adequate trainings, tools and resources		
4.1	What are some of the barriers you face in addressing mental health needs of healthcare workers?		Probes:<br>• Do you think lack of trainings/resources as one of the barriers to addressing mental health needs of healthcare workers?<br>• How health system can address these barriers?
4.2	In your opinion, how these barriers could be handled efficiently, to ensure adequate mental health support for healthcare workers?		
5	**Theme: Suggestions and recommendations to address psychological needs of health care workers**		
	Here I would like to understand how institutions could provide mental health support to healthcare workers. Specifically, we would like to understand the interventions that the health system can offer to alleviate psychological burden of healthcare workers.		
5.1	What kind of relief healthcare workers expect to manage with these pressures?		
5.2	Do you have suggestions on how institutions could provide mental health support services for coping with stress related to this pandemic		Probes:<br>• How health system is prepared to address psychological needs of healthcare workers?<br>• How health system can ensure implementation of process and tolls to support mental health needs of healthcare workers?<br>• How health system will sustain the mental health support activities for its healthcare workers?
5.3	Do you have suggestions on how provincial/federal government could provide mental health support services for coping with stress related to this pandemic		

For peer review only - http://bmjopen.bmj.com/site/about/guidelines.xhtml

COREQ (COnsolidated criteria for REporting Qualitative research) Checklist

A checklist of items that should be included in reports of qualitative research. You must report the page number in your manuscript where you consider each of the items listed in this checklist. If you have not included this information, either revise your manuscript accordingly before submitting or note N/A.

Topic	Item No.	Guide Questions/Description	Reported on Page No.
**Domain 1: Research team and reflexivity**			
Personal characteristics			
Interviewer/facilitator	1	Which author/s conducted the interview or focus group?	7
Credentials	2	What were the researcher's credentials? E.g. PhD, MD	7
Occupation	3	What was their occupation at the time of the study?	7
Gender	4	Was the researcher male or female?	7
Experience and training	5	What experience or training did the researcher have?	7
Relationship with participants			
Relationship established	6	Was a relationship established prior to study commencement?	6
Participant knowledge of the interviewer	7	What did the participants know about the researcher? e.g. personal goals, reasons for doing the research	6
Interviewer characteristics	8	What characteristics were reported about the inter viewer/facilitator? e.g. Bias, assumptions, reasons and interests in the research topic	N/A
**Domain 2: Study design**			
Theoretical framework			
Methodological orientation and Theory	9	What methodological orientation was stated to underpin the study? e.g. grounded theory, discourse analysis, ethnography, phenomenology, content analysis	7
Participant selection			
Sampling	10	How were participants selected? e.g. purposive, convenience, consecutive, snowball	6
Method of approach	11	How were participants approached? e.g. face-to-face, telephone, mail, email	6
Sample size	12	How many participants were in the study?	6
Non-participation	13	How many people refused to participate or dropped out? Reasons?	7
Setting			
Setting of data collection	14	Where was the data collected? e.g. home, clinic, workplace	6
Presence of nonparticipants	15	Was anyone else present besides the participants and researchers?	7
Description of sample	16	What are the important characteristics of the sample? e.g. demographic data, date	8
Data collection			
Interview guide	17	Were questions, prompts, guides provided by the authors? Was it pilot tested?	6
Repeat interviews	18	Were repeat inter views carried out? If yes, how many?	7
Audio/visual recording	19	Did the research use audio or visual recording to collect the data?	7
Field notes	20	Were field notes made during and/or after the interview or focus group?	7
Duration	21	What was the duration of the inter views or focus group?	7
Data saturation	22	Was data saturation discussed?	7

For peer review only

Transcripts returned	23	Were transcripts returned to participants for comment and/or correction?	6
**Domain 3: analysis and findings**			
Data analysis			
Number of data coders	24	How many data coders coded the data?	7
Description of the coding tree	25	Did authors provide a description of the coding tree?	7
Derivation of themes	26	Were themes identified in advance or derived from the data?	7
Software	27	What software, if applicable, was used to manage the data?	7
Participant checking	28	Did participants provide feedback on the findings?	7
Reporting			
Quotations presented	29	Were participant quotations presented to illustrate the themes/findings? Was each quotation identified? e.g. participant number	7
Data and findings consistent	30	Was there consistency between the data presented and the findings?	NA
Clarity of major themes	31	Were major themes clearly presented in the findings?	8
Clarity of minor themes	32	Is there a description of diverse cases or discussion of minor themes?	8

Developed from: Tong A, Sainsbury P, Craig J. Consolidated criteria for reporting qualitative research (COREQ): a 32-item checklist for interviews and focus groups. International Journal for Quality in Health Care. 2007. Volume 19, Number 6: pp. 349 – 357

Once you have completed this checklist, please save a copy and upload it as part of your submission. DO NOT include this checklist as part of the main manuscript document. It must be uploaded as a separate file.

For peer review only

BMJ Open

Impact of COVID19 on mental health of primary healthcare workers in Pakistan: lessons from a qualitative inquiry

Journal:	*BMJ Open*
Manuscript ID	bmjopen-2022-065941.R2
Article Type:	Original research
Date Submitted by the Author:	16-Nov-2022
Complete List of Authors:	Hameed, Waqas; The Aga Khan University, Community Health Sciences<br>Avan, Bilal Iqbal; London School of Hygiene and Tropical Medicine,<br>Feroz, Anam Shahil; The Aga Khan University, Department of Community Health Sciences<br>Khan, Bushra; University of Karachi, Department of Psychology<br>Fatmi, Zafar; Aga Khan University, Department of Community Health Sciences<br>Jafri, Hussain; Fatima Jinnah Medical University<br>Wassan, Mansoor Ali; Government of Sindh<br>Siddiqi, Sameen; Aga Khan University Medical College Pakistan, Community Health Sciences Department
**Primary Subject Heading**:	Mental health
Secondary Subject Heading:	Mental health, Public health, Qualitative research
Keywords:	MENTAL HEALTH, HEALTH SERVICES ADMINISTRATION & MANAGEMENT, COVID-19

SCHOLARONE™ Manuscripts

I, the Submitting Author has the right to grant and does grant on behalf of all authors of the Work (as defined in the below author licence), an exclusive licence and/or a non-exclusive licence for contributions from authors who are: i) UK Crown employees; ii) where BMJ has agreed a CC-BY licence shall apply, and/or iii) in accordance with the terms applicable for US Federal Government officers or employees acting as part of their official duties; on a worldwide, perpetual, irrevocable, royalty-free basis to BMJ Publishing Group Ltd ("BMJ") its licensees and where the relevant Journal is co-owned by BMJ to the co-owners of the Journal, to publish the Work in this journal and any other BMJ products and to exploit all rights, as set out in our licence.

The Submitting Author accepts and understands that any supply made under these terms is made by BMJ to the Submitting Author unless you are acting as an employee on behalf of your employer or a postgraduate student of an affiliated institution which is paying any applicable article publishing charge ("APC") for Open Access articles. Where the Submitting Author wishes to make the Work available on an Open Access basis (and intends to pay the relevant APC), the terms of reuse of such Open Access shall be governed by a Creative Commons licence – details of these licences and which Creative Commons licence will apply to this Work are set out in our licence referred to above.

Other than as permitted in any relevant BMJ Author's Self Archiving Policies, I confirm this Work has not been accepted for publication elsewhere, is not being considered for publication elsewhere and does not duplicate material already published. I confirm all authors consent to publication of this Work and authorise the granting of this licence.

For peer review only

Bismillah hir Rahman nir Raheem

Title:
Impact of COVID19 on mental health of primary healthcare workers in Pakistan: lessons from a qualitative inquiry

Short title: COVID19 and mental health of primary healthcare workers

Authors:
Waqas Hameed[1], Bilal Iqbal Avan[2], Anam Shahil Feroz[1], Bushra Khan[3], Zafar Fatmi[1], Hussain Jafri[4], Mansoor Ali Wassan[5], Sameen Siddiqi[1]

[1]Department of Community Health Sciences, Aga Khan University, Karachi, Pakistan
[2]Department of Clinical Research, London School of Hygiene and Tropical Medicine, London, United Kingdom
[3]Department of Psychology, University of Karachi, Sindh, Pakistan
[4]Fatima Jinnah Medical University, Lahore, Pakistan
[5]Department of Health, Government of Sindh, Pakistan

Correspondence:
Bilal Iqbal Avan
Associate Professor
Department of Population Health
London School of Hygiene and Tropical Medicine
Keppel St, London WC1E 7HT, United Kingdom
bilal.avan@lshtm.ac.uk

For peer review only

For peer review only - http://bmjopen.bmj.com/site/about/guidelines.xhtml

Abstract

Objectives
The existing literature regarding the mental health consequences of COVID19 among healthcare workers resolves predominately around specialised hospital settings, while neglecting primary healthcare workers who are the first point of contact for patients. In view of negligible evidence, this study explored mental health impact of COVID19, health system response, and sought suggestions and recommendations from the primary healthcare workers (PHCWs) to address their mental health needs in pandemic crisis.

Design
We employed a qualitative exploratory design.

Setting: A total of 42 primary healthcare facilities across 15 districts in Sindh and Punjab provinces of Pakistan.

Participants: We telephonically conducted 47 in-depth interviews with health service providers and hospital managers. A combination of inductive and deductive approach was used for data analyses using NVivo version 11.0.

Results
There was immense fear, stress and anxiety among PHCWs of being infected and infecting their families at the beginning of this outbreak and its peak which tapered off over time. It was triggered by lack of information about the virus and its management, false rumours, media-hype, lack of personal protective measures (PPEs), non-cooperation from patients and community people. Awareness raising trainings and PPEs provided by the healthcare system, while emotional support from co-workers and supervisors was instrumental in addressing their mental health needs. Additionally, they recommended appreciation and recognition, and provision of psychosocial support from mental health professionals.

Conclusion
Primary healthcare system should be prepared to provide timely informational (e.g. continuous updates in training and guidelines), instrumental (e.g. provision of personal protective equipment, appreciation and recognition), organisational (e.g. safe and conducive work environment), and emotional and psychosocial support (e.g. frequent or needs-based session from mental health professionals) to PHCWs in order to mitigate the mental health impact of pandemic crisis.

Keywords: COVID19, primary healthcare, mental health, psychosocial, health system

For peer review only - http://bmjopen.bmj.com/site/about/guidelines.xhtml

Strengths and limitations of this study:

- Our study gathered mental health experiences of a neglected group health care workers that belonged to primary healthcare settings
- Unlike urban-focused studies, the participants in this study were invited across fifteen urban and rural districts in two most populous provinces of Pakistan
- Conduction of phone-based interviews due to the restricted mobility during COVID-19 was a key study limitation which may introduce some risk of bias as the interviewers did not have the opportunity to build rapport as in the case of face-to-face interviews

For peer review only

For peer review only - http://bmjopen.bmj.com/site/about/guidelines.xhtml

Introduction:

The novel coronavirus disease 2019 (COVID19) has swept across the world.[1] As of September 28, 2021, 232 million people have been infected by COVID19 and approximately 4.75 million people have lost their lives worldwide[2], including 115,000 healthcare workers (HCWs)[3]. This large-scale unprecedented public health catastrophe, placed the frontline HCWs, who are directly involved with all aspects of care of COVID19 patients, at risk of developing psychological distress and other mental health symptoms.[4] Studies conducted thus far provide evidence of the adverse impact of COVID19 on mental health and well-being of HCWs, primarily those who are involved in providing specialised care to COVID19 patients[5]. According to a systematic review, the pooled prevalence of depression, anxiety and post-traumatic stress disorder among HCWs related to the COVID19 pandemic across 21 (low to high income) countries hovered around 22%[5]. The prevalence of such mental health disorders vary according to sex, age, and role of HCWs[6,7].

While these studies made substantial contribution to inform strategies in alleviating the burden of psychological distress among HCWs, the prime focus has been on professionals who are working in hospital settings and are primarily engaged in the provision of specialised care to COVID19 patients[6,7]. In contrast, primary healthcare workers (PHCWs) have been managing a major share of the burden of COVID19 related care[8] by being the first point of contact for a broad spectrum of COVID19 patients, whether suspected, confirmed (with or without symptoms), or exposed to case(s), as well as those with non-COVID19 health needs[9]. Studies indicate that some 80% of cases are mild and the majority of moderate cases seek primary healthcare (PHC) services as the entry point for getting medical care[10].

Community health workers (CHWs) also played a distinct role during COVID19, in lower middle-income countries (LMICs), by being involved in community awareness, engagement and sensitisation and contact tracing though at the cost of routine service delivery [11]. These newly assigned tasks during pandemic not only exposed them to increased risk of infection, but they also experienced stigmatisation, isolation or social ostracisation[11]. Ironically, they reportedly received inadequate support from health system in the form of personal protective equipment[12], and logistics and supportive supervision. Besides that, PHCWs are also responsible to manage the aftermath of contingencies and bearing the collateral damage caused by reallocating resources to COVID19 whilst inadvertently hindering access to a wide range of primary healthcare (PHC)services[13,14.] This put them under stressful situations during this pandemic[15]. A study from Canada showed that interpersonal care team rapidly shifted their practices to support their patients during COVID19 pandemic; and they experience a surge in mental health issues among their patients[16]. Similarly, a large-scale survey from Canada revealed that COVID19 resulted in increased work-load, loss of employment, redeployment to new setting, concerns for health and safety among social workers at the frontline[17].

Despite the ongoing debate on how strengthening PHC (including CHWs) can help health systems adapt during the COVID19 pandemic[18,19] the significance and instrumental role of PHCWs in the times of COVID19 pandemic has received little attention of the global research community. A large majority of the research that assessed mental health consequences of COVID19 among HCWs has focused in the specialised hospital settings[20]. Furthermore, there are few qualitative studies that provide in-depth mental health experiences of HCWs, in general, as well as those working in PHC settings [21]. Even a recent effort to consolidate potential interventions to address mental health issues of HCWs during such outbreak is largely based evidence generated from HCWs working in specialised settings, while neglecting the mental health needs of and solutions for PHCWs[22].

For peer review only - http://bmjopen.bmj.com/site/about/guidelines.xhtml

Keeping in view of the role that PHCWs as alluded to above, we hypothesised that they may have suffered from similar mental health issues as faced by HCWs working in specialised healthcare settings. Furthermore, as COVID19 is expected to become endemic[23,24], the role of PHC becomes pivotal for its community-based information structure to identify risk groups, monitor compliance, provide preventive and promotional services, and contact tracing in this and similar future pandemic. Hence, their mental health needs, readiness and response become central to tackling such issues. In order to better support PHCWs with their adapted role during COVID19, interventions are needed that could effectively cater to their mental health (which include mental, emotional and social) needs in the long run [25]. With this rationale, we set following research questions for this research that focuses on public-sector PHCWs in Pakistan.

Research questions:

1. What is the perceived mental health impact of COVID19 on PHCWs in Pakistan?
2. How health system has responded to the mental health needs of PHCWs?
3. What are the recommendations to support mental health and well-being of PHCWs in pandemic crisis?

Country Context:

Pakistan has a population of over 225 million inhabitants[26]. The first COVID19 case was identified in February 2020, and since then a total number of confirmed cases exceed 1.2 million along with 27,638 deaths has been registered (till September 28, 2021)[27]. The country has experienced four waves – each with different variant including delta, more recently[27]. With a focus on vulnerable groups of HCWs and elderly (65+ years) people, the country embarked on a nation-wide vaccine campaign for COVID19. The vaccine is now available for everyone except for children under the age of 12 years[27]. While several vaccines are safe and effective, there is strong emphasis on practicing preventive measures by the government.

Public-sector healthcare in Pakistan is delivered through a three-tiered health system. The PHC provides services through a community-based component comprising over 100,000 CHWs and health facility component that includes almost 10,000 basic health units (BHUs), rural health centres (RHCs), maternal and child centres (MCH), and civil dispensaries[28]. On average, a BHU and RHC covers a population of 25,000 and 80,000, respectively. These static primary health care facilities are supplemented by approximately 100,000 community health workers (referred to as 'lady health workers')[29]. The government of Pakistan has taken several measures to combat this deadly pneumonia virus[30] but it leaves a lot to be desired[31]. The health system of Pakistan is weak[32] and with critical shortage of health workforce, and is not prepared to sustain a major surge in COVID-19 cases[33]. Few studies have been conducted to assess the impact of COVID19 on mental health of HCWs; while these studies do identify symptoms of mental disorders, all have focused on tertiary level of health system [34-36].

Methods:

Study design and setting

A qualitative exploratory design was adopted using in-depth interview technique. Exploratory design enabled us to gather detailed description of HCWs experiences during the COVID19 pandemic. The study was conducted in 42 health facilities across 15 districts of Sindh and Punjab provinces of Pakistan. Districts where the cumulative number of COVID19 cases were high since the onset of this outbreak till date of survey were selected. Participants were selected using a criterion purposive sampling approach from randomly selected public health facilities stratified by districts and type of health facilities (BHUs and RHCs). We also collected contact details of affiliated Lady Health Workers[29] from the in-charge of the selected health facility.

For peer review only - http://bmjopen.bmj.com/site/about/guidelines.xhtml

Recruitment of study participants:

The study participants were classified into three groups: lady health workers (LHWs), facility-based health service providers (doctor, nurse, midwife, lady health visitor), and administrators/managers (medical superintendent, lady health supervisors). LHWs are attached to a local health facility, but they are primarily community-based, working from their homes. They serve the whole community, but they play a particularly important role in maternal and child health care in rural and urban slum communities by coordinating efforts with traditional birth attendants and midwives[37]. These LHWs work under the supervision of lady health supervisors[38,39]. In contrast, the facility-based healthcare workers are based at PHC facilities and they provide following services: general out-patient services, antenatal care, deliveries, postnatal care, and vaccination. In addition of these, diagnostic services (lab, x-ray) and dental outpatient services are also provided in RHCs. The PHCWs were primarily involved in awareness raising and contact training regarding COVID19. Some of the facility-based staff were also seconded to COVID19 isolation centres. Even though administrators at the PHC also often directly involved in service provision, we kept this distinction to see any diversity in their responses as opposed to their counterparts. The study participants were recruited after obtaining formal permission from the higher government authorities, by contacting administrators of the randomly selected health facility.

Data collection:

A standard semi-structured guide was used to interview all study participants, which was developed following extensive review of literature and consultation. It comprised of three major themes: a) Impact of COVID-19 on mental health and well-being of health care professionals; b) Mechanism available within healthcare system to address mental health needs; and c) Mental health needs of PHCWs, and suggestions and recommendations to address their needs. Open-ended questions and probes encouraged free flow of information from participants to developed deeper understanding of their experiences. The interview guide was thoroughly reviewed and discussed by the core research investigators (WH, BIA, ZF, AF, NA, BK and SSQ).It was then pre-tested and revised based on few PHCWs in districts that were included in the larger quantitative survey[40,41,42]. During pre-testing, no major current challenges were reported by most PHCWs since the first wave has already passed[42]. Therefore, the guideline was revised to gather their experiences from the onset of COVID19 to current situation, and getting insights on how situation evolved over time. (see supplementary file 1)

The data collection took placed between August and October 2020. Owing to restricted mobility due to COVID19, a total of 47 in-depth interview (IDIs) (Sindh: 29; Punjab: 18) were conducted telephonically until data saturation was achieved[43]. In the first phone call, participant was informed about the study and scheduled an appropriate data and time for the interview, if the participant is busy. Subsequent call was made at the schedule time for the interview. Experienced data collectors with a background in sociology or psychology were trained to conduct the interviews in local language (Urdu). All the calls were made from the Aga Khan University under close supervision of study investigators. Data analysis was concurrently performed to determine the point of saturation. On average, the interviews lasted for 25-35 minutes. With the consent of participants, all the interviews were audio-recorded on tablets using a built-in call recording feature in the tablets. It was ensure that participant is comfortable during the interview and no one is sitting near to them whose presence could possibly influence his/her responses. Data collectors were also encouraged to maintain key interview notes. A linguistic expert later transcribed the recordings into English for analysis. The transcripts were not returned to the participants due to

For peer review only - http://bmjopen.bmj.com/site/about/guidelines.xhtml

their busy schedule. Five participants refused to participate in the study; they were mainly the health managers due to their busy schedule. No repeat interview was conducted. To ensure data quality, a few initial interviews were conducted by WH (PhD scholar – health system) and AF (PhD scholar – public health) in presence of trained data collectors. Both the authors were working as a university faculty at the time of the study and had vast experience of conducting such interviews.

Data analysis:

All interview notes, recordings, and transcriptions were imported into the NVivo version 11.0 software for analyses, with no identifying characteristics included in the transcriptions[44]. We explored divergent and congruent views of three groups, that is, facility-based service providers, administrators, and lady health workers. Conventional content analysis technique was used to inductively analyse all data[45]. Two independent researchers (WH and ASF) applied inductive coding while reviewing the transcriptions in NVivo[44] and identified new emerging codes and categories. The codes and themes were compared and the any discrepancies in the interpretation of codes and themes were discussed among the investigation team to reach a consensus. The discrepancies were discussed in the large team meeting until agreement on the themes was achieved. In situations when it was difficult to achieve consensus among the team, senior author facilitated discussions and helped obtain agreements among the research team members. In order to gain a more comprehensive understanding of mental health impact, health system response, and suggestions and recommendation to support their mental health needs, sub-themes were reviewed and compared by additional reviewers (BK and ZF) to examine corroboration and convergence. Independent review of data and coding allowed coherent interpretations that are grounded in the data[41] We did not obtain feedback from the participants on study findings.

Patient and public involvement

We sought inputs from target audience through pre-testing of interview guides to ensure comprehension of the questions. Furthermore, in consultation with provincial health departments we selected districts where COVID-19 cases were high. The focal person in each selected district was oriented about the study who connected our team with in-charge of health facility to avoid any miscommunication. Lastly, they also advised about the dissemination of research findings – that is, they specifically emphasis to share it through research briefs to reach wider audience in the country.

Ethical considerations

Due to the telephonic interview, verbal informed consent was taken from all study participants which included permission to audio-record their interviews and use anonymized quotes. All study participants were informed about the study purpose, their rights as study participants, confidentiality, and publication of study findings in anonymised manner. We shared official permission letters with study participants issued by respective health department of government of Punjab and Sindh provinces to build trust. Ethics approval was obtained from Aga Khan University (Ref. number: 2020-5186-11602) and National Bioethics Committee of Pakistan (Ref. Number: 4-87/COVID-45/NBC/20/393).

Consolidated criteria for reporting qualitative research (COREQ) guidelines were used to organise this paper[46]. (see supplementary file 2)

Results:

For peer review only - http://bmjopen.bmj.com/site/about/guidelines.xhtml

A total of 47 IDIs were included in the analysis. Table 1 shows the socio-demographic characteristics of participants. The mean age was 43.2 (±10.9) years and had an experience of 14.4 (±9.4) years. Approximately half were female (44.7%), 55.3% were health managers, and about 51.1% were working at or affiliated with the Basic Health Unit. The in-charge of health facilities (BHU and RHC) were all medical doctors and mostly males.

Table 1: Characteristics of study participants

Characteristics	n (%)
**Sex**	
Female	21 (44.7)
Male	26 (55.3)
**Age in years**	
Mean (±SD)	43.2 (10.9)
**Designation**	
Health service providers (doctor, nurse/midwife)	16 (34.0)
Health managers (lady health supervisors – supervises LHW, in-charge of health facility)	26 (55.3)
Lady Health Workers (LHWs)	5 (10.6)
**Type of health facility**	
Basic Health Unit (BHU)	24 (51.1)
Rural Health Centre (RHC)	23 (48.9)
**Total experience in years**	
Mean (±SD)	14.4 (9.4)

The main study findings are organised into three main themes: perceived mental health impact of COVID-19; support mechanisms to address their mental health needs; and suggestions recommendations made by PHCWs. Where observed, we also presented contrasting views of service providers and health managers, highlighting the diversity in provincial perspective (table 2).

Table 2: Themes and Categories

Themes	Categories
Theme 1: Perceived mental health impact of COVID19 on healthcare workers	1.1 Anxiety due to uncertainty of Covid-19 and media exaggeration<br>1.2 Fear of infection, isolation and stigma<br>1.3 Challenges regarding patients<br>1.4 Stress due to lack of availability of personal protective equipment<br>1.5 Stress due to excessive work-load
Theme 2: Available support mechanisms to address mental health needs of healthcare workers	2.1 Awareness raising of healthcare workers<br>2.2 Provision of personal protective equipment<br>2.3 Social support from co-workers and family members<br>2.4 Managerial support from supervisors
Theme 3: Mental health needs of healthcare workers and recommended solutions	3.1 Safe and conducive work environment<br>3.2 Awareness about evolving situation and corrective actions<br>3.3 Psychosocial support from mental health professionals<br>3.4 Appreciation and recognition of their work in the pandemic crisis

1. Mental health impact of COVID19 on healthcare workers

For peer review only - http://bmjopen.bmj.com/site/about/guidelines.xhtml

1.1 Anxiety due to uncertainty of COVID19 and media exaggeration

All study participants reported experiences of immense fear and anxiety due to the uncertainties around COVID19 at the onset of this outbreak in Pakistan. It was primarily triggered by lack of knowledge, prevalent misconceptions about COVID19, and being aware of its disastrous effect in the developed world. There was a perception that it was a fatal virus and whoever got it would die. Others were sceptical about its existence like how long will this outbreak last and will they remain bound forever to the new operational changes in service delivery.

"At the beginning, the real fear among people was that - a rumour spread that, if God Forbids, this much dangerous disease, and someone gets it he will die". Health manager, Ghotki.

"There's a bit of worriedness, mental pressure, what to do what not to do, how to go, should we go to their house or not, these problems happens, mental stress occurs" Lady Health Worker, Jamshoro".

For many HCWs was not being able to distinguish between COVID-19 positive and negative people around them due to its asymptomatic characteristics and lack of testing facility was mentally disturbing. It made the HCWs reluctant to interact with patients and community members, and even with their colleagues at the very beginning of the outbreak. This was consistent among facility-based service providers, health managers and lady health workers.

"We ourselves were also mentally disturbed, that how do we know, we are going on duties, on fields, we staffs are meeting each other so how do we know that who has corona symptoms and who does not". Lady Health Supervisor, Rawalpindi

1.2 Fear of infection, isolation and stigma

While most of the PHCWs were not directly involved in providing care to COVID19 patients, there was still some fear of being contracted with COVID19 during their interaction with patients at the beginning and during peak time. Similar fear existed among LHWs during their routine household and targeted visits for contact tracing.

"We were afraid that we might get infected or our families might get infected. So we spend all this time under stress. We were seeing that there were many positive cases in our neighbourhood and the news on media". Health Manager, Shaheed Benazirabad

Some of the facility-based staff was seconded to COVID19 isolation centres. It was highlighted that the level of fear was considerably higher among them. Similar, the level of fear possible transmission was higher among HCWs who had any chronic disease (e.g. diabetes) or had old parents or young children, be they work at the health facility or in the community.
"At that time, my female health workers were very disturbed mentally. She was like we have small kids how we will run out-patient services… how everything will be managed. Their families were also tensed that you are going on duties and you are putting us on danger as well". Health Manager, Shaheed Benazirabad

These stressors and uncertainties of being infected also affected provider-patient interaction during the initial phase of the outbreak and as it peaked. A service provider shared that:

For peer review only - http://bmjopen.bmj.com/site/about/guidelines.xhtml

"When a person is mentally unwell and is feeling uncertain, then s/he wouldn't even deliver what is normally expected of him/her. We were not able to take care of the patients then. We didn't know whether the incoming patient is COVID positive or not. We just kept a distance and asked patient ailment. We had to save ourselves someway". Service Provider, Faisalabad

Fear of isolation was not reported by many HCWs. Only a few facility-based service providers reported to be stressed as they had to stay away from their family members, or by being alone.

"She [my co-worker] took her medicine regularly for 14 days at home. Then her [COVID19] test was conducted after 14 days and it came negative. But still she was very worried and tensed, and she was crying that she had to stay away from her kids". Service Provider, Khairpur

Mixed responses were reported by the LHWs in terms of stigma faced by them. According to some LHWs, community members resisted their entry into the households; whereas, senior or experienced LHWs reported fewer such issues in the field.

"During corona [peak] days, when we [I and my LHW] used to go out, people used to complain a lot. A huge pandemic has spread and you are coming to our house, so how do we know whether you have it [COVID19] or not; you can infect us. We felt this kind of resistance a lot in field even though we used to go with PPEs (sanitizers and masks)… at some places, sometimes it [people's reaction] was normal and some people gave very positive responses". Lady Health Supervisor, Rawalpindi

1.3 Challenges regarding patients:

A major challenge that was a source of stress and frustration among HCWs was uncooperative patients. Facility-based staff were concerned that patients were reluctant to wear face-mask or to adhere physical distancing within the premises of health facilities, and at times, they became offensive when they were insisted to take protective measures for COVID19.

"Our stressor was our patients because they were not ready to understand the situation. They didn't cooperate with us. They thought we didn't want to treat them. We get the announcement done here in our local areas and also pasted the posters. We also wrote on the board outside to 'wash your hands' and 'wear mask in hospital'; but we had to tackle a lot of troubles like these. Health Manager, Gujrat

Due to illiteracy, people did not believe in the existence of any viral outbreak, and they considered such protective measures as tactics of health workers to avoid service delivery.

"Only those women are worried who are a little bit educated but mostly aren't, 99% of people are don't have any fear and they don't believe in corona existence and they think that we are making them stupid". Health Manager, Mirpur Khas

Early on, another challenge faced by facility-based staff (both service providers and managers) was to conduct COVID19 test of suspected patients. According to them, people were extremely reluctant to share any details or willing to undergo COVID19 test. This was primarily due to the prevalent myths and misconceptions regarding COVID19 at the beginning of this outbreak.

"People used to bring sticks to beat us because it is a rural area and villagers used to say that our patients didn't have anything and they asked us to go away. We had to call assistant commissioner of that area than he used to send police. After that we were able to take samples

For peer review only - http://bmjopen.bmj.com/site/about/guidelines.xhtml

from them. It was mental torture for us. Believe me, when we tell people that we are about to take their samples, tell us your mobile number and Identification Card number etc. I swear they curse us and run away" Service Provider, Sargodha*

1.4 Stress due to lack of availability of personal protective equipment

Lack of availability of personal protective equipment or supplies put both facility- and community-based HCWs under immense stress of being infected. Even facility-based HCWs who were seconded to a quarantine centre expressed concerns about unavailability of PPE.

"I had duty in quarantine for 3 months then I didn't have any kit or PPE, or mask or glove. I got a call and they told me to come on duty that day at 2 so I started crying because both my parents were home. We heard about doctors and XYZ deaths. I was scared and didn't have any protection kit". Service Provider, Sialkot

In the absence of PPEs, some of the facility-based service providers and lady health workers made use of the scarf or linen to cover themselves and their patients' face or a community member during household visit, respectively; while majority them reported to have it purchased on their own.

"Obviously, there weren't many masks available for us even and for patients. So any piece of cloth, or scarf, we use to make them cover their mouths and nose. Males sometimes used linen or face mask". Health Manager, Ghotki

1.5 Stress due to excessive work-load

Increased work-load was mainly the concern of field-based staff (LHWs and LHS) and for those who were given additional responsibilities like secondment to COVID19 isolation centre. Field teams had to locate suspected cases at a short notice. Due to lack of proper transportation, especially during lockdown, made things were quite stressful and exhaustive.

"Everybody was busy running to and fro because [COVID19] cases would come without notice. Doctor would get anxious and ask us to collect data and pictures [of patients]. We had to go to LHS and LHW. Lady Health Supervisor, Rawalpindi

On the contrary, the work load of facility-based service providers reduced considerably during the initial phase in the health facilities as people was reluctant to go to health facilities in the fear of COVID and certain prevalent myths.

"Yes, it (health facility) was working but patients were very less. Usually we have 200 or 300 patients but during corona we had only 30 or 40 patients. Even there was news circulating in news channels that poison injection were being given in the hospitals to corona patients. So people were really afraid and due to these kinds of rumours they stopped coming to hospitals". Service Provider, Sargodha

2. Available support mechanism to address mental health needs of health workforce

2.1 Awareness raising of healthcare workers

All facility- and community-based HCWs reported to have received awareness raising trainings that were organised by the department. The trainings primarily focused on preventive aspect of COVID19: characteristics of virus, signs and symptoms, modes of transmission, population at

For peer review only - http://bmjopen.bmj.com/site/about/guidelines.xhtml

risk, preventive and protective measures, quarantine guidelines, and its testing.

"The purpose of training was to screen yourself, keep yourself safe, save the people of your organization, and also protect community; things that can control the disease. If a patient has symptoms, tell them where to do test and how it is done". Service Provider, Khairpur

However, participants from all three study groups unanimously informed that they had not received any training on mental health except for one lady health supervisor.

"In trainings, we talked on all issues, right. But, the main issue that was not touched is people's mental condition during corona. Nobody ever tried to assess that amongst us. We have never ever tried to understand people mental level or their mental condition or our feelings". Lady Health Supervisor, Rawalpindi.

According to all HCWs, increased awareness and declining COVID19 cases over time mitigated the fear and stress among healthcare workers.

"Right now [health] workers are not depressed they are normal and doing their routine work. They were depressed and worried in the beginning but now they are normal due to awareness campaigns". Health manager, Shaheed Benazirabad

2.2 Provision of personal protective equipment

The PPEs were provided to the health workers with some delays, after few weeks of the outbreak in the country. The use of these PPEs, combined with other protective measures of COVID19, played a major role in reducing fear and stress among healthcare workers.

"We displayed the posters outside of hospitals - informing people not to come to the hospital without any reason. Maintain social distancing, wash hands, make soaps available everywhere and made hand sanitizers available. That's how we coped up with the stress. To be very frank, in the beginning everything was very difficult for me and for them [health workers]". Service Provider, Multan

"Firstly, protection, masks, shields etc. there was a shortage before but now they are available. There is also awareness now, we are seeing that there are fewer patients in Pakistan compared to other countries. All these things are effective for mental health". Service Provider, Sialkot

2.3 Social support from co-workers and family members

Provision of social support by co-workers - including colleagues and supervisors – emerged as a common response mechanism to mitigate the fear, stress and anxiety among both facility- and community-based HCWs. During the early phase of this outbreak when stress, anxiety and fear was high, staff members used to discuss and provide social support to each other. Senior HCWs often used to enquire about challenges being faced by their colleagues, including if they are emotionally disturbed. They used to console them to help overcome any kind of fear, stress or anxiety pertaining to COVID19.

"We just provided them emotional support. I educated staff members that we are health care providers, how could we help people if all of us become a victim of mental health issues. We have to be strong because we are on frontline. We don't have to be fearful. Along with that we used to

For peer review only - http://bmjopen.bmj.com/site/about/guidelines.xhtml

appreciate workers. What if they themselves become depressed then who would work. I told them that corona has no limit so we will have to live with it". Health Manager, Khairpur

A couple of HCWs acknowledged that they used to consult and take frequent advice from ex-physiatrist mates to overcome stress, and some received support from their family members.

"I was in contact with a psychiatrist so in that way I used to get constant help from there. I personally took many sessions". Service Provider, Faisalabad

"My husband kept on telling me that the night that is in the grave is not outside, so stop thinking about it and the major support that my husband gave me was it is a death by martyrdom so I used to get relaxed from that as well". Lady Health Supervisor, Rawalpindi

The emotional support was provided in several ways. Some of the consoling approaches were: a) reinforcement of the use of PPEs and COVID19 SOPs; b) reiterated the purpose of being a health worker; c) ensured frequent communication with the stressed person and giving assurance of being with them; d) motivated colleagues by giving their own example; e) encouraging others to keep themselves busy if they are stressed. Importantly, some used religious healing perspective by linking everything with God 'Allah' that He will take care of everything.

"All you have to do is care, and this is a type of help and if any patient gets well with our help it would be a blessing from Allah, living and dying is Allah's decision, we can only take care so take care and help others, you will be rewarded InshaAllah [if Allah wills] so we say all this to each other and we give support to each other". Service Provider, Dadu

"I was continuously in contact with her [COVID19 positive health worker] and tell her to take care of herself and she was telling her children whether test will come negative or not. She was scared that she had corona and she would die. Yes, we told her God will help you. We have doctors with us and our district manager and medical superintendent are with us, they were talking to her continuously. They were also consoling here that corona will end why you are worried?" Service Provider, Khairpur

"Reassuring, giving trust, and with positive thinking for the future, telling them that we are messengers, Allah has placed us to serve these people, to take care of them, to tell them preventions". Lady Health Supervisor, Rawalpindi

A few facility-based HCWs kept themselves busy in extra-curricular activity to deal with their stress like reading or even social media.

"I kept myself engaged because whenever I was free then I would get a lot stress when you hear news etc. I was studying and spent most of my time with my co-worker. And whenever I would go home, I would keep myself engaged mentally". Service Provider, Sialkot

2.4 Managerial support from supervisors

According to a majority of service providers and lady health workers, their supervisors/managers provided a lot of support in managing work-related issues that were causing stress. The supervisors primarily managed issues of their teams by themselves, or at times, by engaging a higher (district) administrative health authorities. Effective communication with team that included constant motivation and awareness raising was instrumental in addressing day-to-day challenges. At some instances, health managers also offered intermittent breaks to the health

For peer review only - http://bmjopen.bmj.com/site/about/guidelines.xhtml

workers who were unwell or became exhausted due to work. A couple of respondents also mentioned that the health facility was closed for a few days when the COVID19 positive staff was quarantined.

"We fulfil their demands, if there is a salary issue, if they haven't received their salary from 2 to 3 months, we write it to DHO that they will not do work with interest so they should be given their salary on time and they forward it to upper department and their problems are solved. Service Provider, Dadu

"I counsel them that see this is difficult for us; you cooperate with me and I will cooperate with you. If you have any problem then you can tell me and take off. If there is no issue then take half day off but come to work next day. So I tried to make them relax like this". Health Manager, Karachi

3. Mental health needs of healthcare workers and recommended solutions

3.1 Safe and conducive work environment

Safe working environment was identified as one of the prominent needs of both facility-based and community-based HCWs. It primarily meant timely provision of PPE kits that should include gloves, face-masks, and hand sanitizers, and provision of security especially to deal with aggressive patients.

"We should have received PPE kits in the beginning before [COVID19] peak time. When cases were increasing in China, since then we should have made ourselves prepared for this". Service Provider, Multan

"Security should be given especially in this COVID situation. Because when patients come to the health facility and the doctor fails to examine them then they create a scene or become aggressive, and misbehave". Service Provider, Sialkot

Primarily, facility-based HCWs who were assigned additional responsibilities related to COVID19 expressed a dire need of intermittent breaks under certain circumstances – for example, when a worker is ill, exhausted due to increased work-load, or experiences any kind of stress.

"If someone appears COVID-19 positive then that person is placed in 14 days quarantine. So if he is already in a stressful condition, his mental health isn't able to hold so at least until he is normalized, there should be some relief given". Service Provider, Faisalabad

"They [health workers] say that how can we perform our normal duties and COVID ward both simultaneously? So they should be given holidays. Our demand is that our staff should be given more holidays and good packages and some financial bonuses should be provided". Health Manager, MirpurKhas

3.2 Awareness about evolving situation of COVID19 and corrective actions

A majority of facility- and community-based HCWs expressed the need for having updated information about the evolving situation regarding COVID19. They also identified the need of

For peer review only - http://bmjopen.bmj.com/site/about/guidelines.xhtml

receiving timely guidelines about the corrective actions to deal with constantly changing situation.

“There should have been sessions about the forthcoming epidemic. Like this what is this disease, what are its consequences, how to deal with it. There should’ve been such learnings that they prepare their workers. They just said that ‘wear masks’, and what’s being told on TV”. Mealth Manager, Khairpur

3.3 Psychosocial support from specialized mental healthcare workers:

Even though social support emerged as a key response-measure used by HCWs to cope with the prevailing stress, getting psychosocial help from specialized mental health professional was still identified as a need and future recommendation by majority of the study participants. Various suggestions were given to have such support in-place for the HCWs, which included: a) having a full-time specialist based district or sub-district level to provide needs-based support, b) conduction of periodic sessions on mental health, c) mobile team of psychologist to provide periodic support of health staff; and d) making mental health component part of the COVID19 trainings.

“Mental health is a drawback here. There should be a proper person, who should, first of all, educate the staff, that how will you improve the mental health of the people. Then, some training should be arranged there, meaning, there should be a proper person sitting there, right, like some psychiatrist, or some people like these”. Lady Health Supervisor, Rawalpindi

“In my opinion, CEO of our hospital should depute someone from DHQ like 3 sessions in a week so that they can give sessions to healthcare workers, perform counselling and try to know their inner problems due to which they are disturbed so that their problems can be solved. And provide them relief if it is related to salary or any other issue”. Service Provider, Sargodha

Some facility-based HCWs suggest use of mobile application to provide information about mental health and well-being.

“They should conduct seminars and this is the era of multimedia everyone has mobile and there should be more content on multimedia and social media about mental health so that we become mentally strong and cater things properly. There should be seminars and sessions and government should launch an app regarding this so that we get information from it regarding mental and physical health and keep on updating it”. Health Manager, Gujrat

On the other hand, facility-based healthcare workers expressed the need of capacity building to provide adequate psychosocial support to co-workers and to their patients, as needed. These trainings are also expected to help them guide general patients or community people to follow COVID19 SOPs.

“No, patients aren’t believing, that’s why psychiatrist is important and there should be training provided by the psychiatrist. Our staff should be provided psychological training, we should know what they [patients] are saying and what their opinions are so then we can handle them accordingly”. Health Manager, MirpurKhas

3.4 Appreciation and recognition of their work in the pandemic crisis:

Both facility- and community-based HCWs demanded recognition and appreciation for the additional work that they had to do during COVID19 crisis. This need was particularly highlighted

For peer review only - http://bmjopen.bmj.com/site/about/guidelines.xhtml

by those who were given additional responsibilities like secondment at isolation centres, contact tracing, and COVID19 testing of suspected cases. This recognition was demanded in the form of monetary incentive for risking their lives, or a letter of appreciation by the health department, or a continuous encouragement from their administrative leadership.

"Lady Health Workers are already doing extra work, putting their lives in danger, should have gotten some extra benefits for this, even if they had gotten any certificate. Even if we get one praise, they want to sacrifice everything". Lady Health Supervisor, Rawalpindi

"I would say that government should facilitate the doctor, increase the allowance, give them a complete personal kit, if someone dies, government should support their family, so this should be on government level, so doctors also work motivated… I would want that government should give full support, financially and morally". Service Provider, Khairpur

Discussion:

The current body of existing literature revolved predominantly around the mental health and well-being of HCWs who are involved in provision of specialised COVID-related health services. It largely neglects the PHCWs who are the first point of contact for patients and are also involved in wide range of COVID19-related activities, which in turn can impact their mental health. Our study is unique that it explored the mental health impact of COVID19, health system response, and sought suggestions and recommendations from the PHCWs to address the mental health needs of PHCWs in pandemic crisis.

Reportedly, constant fear, anxiety and stress of being infected and infecting others was immense at onset of COVID19 outbreak and during its peak time. This was primarily triggered by the lack of information about the virus and its management, false rumours regarding COVID19, media-hype, lack of availability of PPEs, and not being able to distinguish between COVID19 positive and negative patients. Evidence show that exaggeration by media tend to negatively affect the mental health of HCWs [47,48]. Similarly, lack of knowledge about COVID19 infection is likely to increase anxiety/depression among physicians[49] and inadequate precautionary measures in the workplace causes fear and anxiety among HCWs[50].

Another important concern causing distress and frustration among healthcare workers was non-compliance of COVID19 SOPs by incoming patients and their companions. According to service providers, low literacy in rural populations, lack of awareness and non-serious attitude towards COVID19 were the primary reasons for this non-adherence. This is consistent with another study from Pakistan that identified non-compliance of COVID19 SOPs as one of the reasons for stress and anxiety among HCWs[35]. Misconceptions and stigma attached to COVID19 also made it immensely challenging for HCWs for contact tracing and COVID19 testing on suspected patients. In extreme cases, people became aggressive and reacted violently to HCWs. Similar incidents have been reported in Pakistan and other countries [51,52]. Such incidents have been associated with work-related stress, burn out, and post-traumatic stress disorder, which in turn negatively affect job performance and quality of services [53].

The mental health impact of COVID19 tapered off over time among PHCWs. PHCWs attributed the reduction of stress and anxiety the support provided by the government in the form of awareness raising sessions regarding COVID19 and its preventive measures, and provision of PPE. More importantly, social support by co-workers (including supervisors) emerged a key factor that alleviated the level of stress and fear among healthcare workers at the beginning when it was very high. Social support by co-workers has also been reported as an effective response-

For peer review only - http://bmjopen.bmj.com/site/about/guidelines.xhtml

mechanism to deal with prevalent mental health issues among HCWs COVID19 crisis [54,55]. Given its significance, fostering a systematic mechanism of social support within healthcare system has also been recommended to safeguard mental health of HCWs during the pandemic[55]. Consistent with other studies [9], patient visits at PHCWs significantly reduced due to the fear of COVID19 infections among general population. Work-load was only a concern of PHCWs were assigned additional task regarding COVID19, and these HCWs reported experiences of exhaustion due to additional responsibilities. They also demanded recognition and appreciation in the form official appreciation letter or monetary incentives for risking their lives. This finding is consistent with other studies where HCWs expressed similar needs [18,51].

In the end, PHCWs rendered few recommendations to address their mental health needs in pandemic crisis. Firstly, they expressed the necessity of having a safe and conducive working environment that included timely provision of PPE for all staff members, safety and security of HCWs to deal with difficult patients, and intermittent breaks – especially for those who are assigned additional COVID19-related responsibilities. The positive effect of safe and conductive work environment on mental health is already discussed above. Second recommendation was around preparedness of HCWs through continuous awareness raising and corrective actions about the evolving pandemic situation. Increased knowledge is also linked with reduced level of stress and anxiety among healthcare workers[49]. Thirdly, they highlighted the need for psychological counselling from mental health professionals and suggested different approach to fulfil this need. Call centres have been established in many countries – including Pakistan[56] - to provide needs-based psychosocial support to HCWs. In Pakistan, this programme perhaps need to expand their scope and provide more targeted services to the PHCWs. Additionally, HCWs requested the need for training to be able to provide psychosocial support to co-worker and dealing with difficult patients or people in the communities. There has already been a debate about potential role of community health workers in the provision of psychosocial support to people during pandemic crisis [57]. The existing "WeCare" programme of the Pakistani government could also be effectively integrated with PHC settings to assist HCWs in providing support to the incoming patients. Lastly, a key need identified by the HCWs, especially those who were assigned additional tasks related to COVID19, was appreciation and recognition for their work in pandemic crisis. They particularly stressed on developing a formal mechanism for the acknowledgement of their efforts via appreciation letter or in the form of monetary incentive or risk allowances. Similar need was also identified in other studies as well [58].

Strengths and Limitations:

In terms of strengthens, our study used gather experiences of a neglected group HCWs that belonged to PHC settings. It uses an inductive interpretative approach to achieve study objectives by gathering information from diverse group of service providers and health managers, including lady health workers. Importantly, this research revealed the perspective and experience of HCWs from the rural settings which are largely neglected in the current body of knowledge. It allowed researchers to look for converging and diverging lines of inquiry to identify common themes/ concepts and incongruence between data sources. On the contrary, a major limitation was that all interviews were conducted over phone which may have constrained rapport building with participant and obtain no-verbal cues during the interviews. In addition, the interviewees might have been distracted during interviews by the activities in their environment and might have experienced fatigue due to long telephonic interviews. Another limitation was that the study was conducted with a focus on public sector hospitals in only two provinces of Pakistan (Sindh and Punjab), which would limit the transferability of the findings to other provinces[59]. However, this study may provide insights for similar PHC settings across Pakistan that are interested in understanding the mental health impact and mental health needs of health service providers

For peer review only - http://bmjopen.bmj.com/site/about/guidelines.xhtml

during Covid-19. Lastly, the study was unable to carry out member checking with study participants, which may have affected the validity of study findings.

Conclusion:

Our study concludes that the stress and anxiety among primary healthcare workers was immense at the beginning of this outbreak or when the COVID19 transmission was at its peak. However, it tapered-off as they become more aware of the virus, receive PPEs, and number of COVID19 cases decreased in their community. Mental health of either health worker or patients were not covered in the trainings offered to them – neither had they had access to any professional psychosocial support. Our study identified a need for a comprehensive support that should be provided to PHCWs in a timely manner, including informational support (e.g. continuous awareness about evolving situation and corrective actions); safe and conducive work environment (e.g. provision of personal protective equipment, personal security to deal with violent reaction of community people due to stigma); organisational support (e.g. intermittent breaks, appreciation and recognition in the form of appreciation letters or monetary incentives (especially for those who are assigned additional responsibilities); and frequent professional psychosocial support to cope with stress but also capacity building trainings on mental health that will enable them deal with difficult patients or community people.

Contributions:

WH and BIA conceptualised the study with inputs from BK, ZF and SS. WH, BIA, BK, ZF, and ASF developed interview guide. HJ and MW facilitated in conducting the study. WH and ASF performed data analysis. BK and ZF reviewed the themes and sub-themes again. WH took the lead in writing the manuscript. All authors critically reviewed the manuscript and provided feedback in shaping the write-up, analysis and presentation of results.

Conflicts of interest: None

Funding: This work was co-funded by the Aga Khan University, Pakistan grant number 20051 and the World Health Organization grant number 202568710-1

Data sharing: The data will be available upon request

Ethics: Ethics approval was obtained from Aga Khan University (Ref. number: 2020-5186-11602) and National Bioethics Committee of Pakistan (Ref. Number: 4-87/COVID-45/NBC/20/393).

Acknowledgements: We are grateful to our implementing partners: Department of Health, Government of Sindh; Primary and Secondary Healthcare Department (P&SHD) and Specialized Healthcare & Medical Education Department from Government of Punjab for extending their full their support and guidance to conduct this survey. We are also thankful to all our study participants for sparing their precious time and made themselves available for the interviews. Last but not the least, we thank our data collectors and project management team: Zafar Dehraj, Imran Sheikh, Sajid Brohi, Noreen Afzal and Ghani Muhammad who managed the implementation of this study in a proficient manner during the pandemic.

For peer review only - http://bmjopen.bmj.com/site/about/guidelines.xhtml

Disclaimer:

The views expressed in this manuscript are those of the authors and contributors, and do not necessarily reflect those of the World Health Organization, or the organization to which the authors are affiliated.

For peer review only

For peer review only - http://bmjopen.bmj.com/site/about/guidelines.xhtml

Reference List

1. Li Q, Guan X, Wu P*, et al.* Early Transmission Dynamics in Wuhan, China, of Novel CoronavirusΓÇôInfected Pneumonia. *New England Journal of Medicine* 2020; 382:1199-1207.https://doi.org/10.1056/NEJMoa2001316

2. Center for Systems Science and Engineering. Coronavirus Resource Center. 27-4-2020. Baltimore, United States, Johns Hopkins University. 27-4-2020.
Ref Type: Online Source

3. World Health Organization. Health and Care Worker Deaths during COVID-19. 20-10-2021. Geneva, World Health Organization. 20-9-2021.
Ref Type: Online Source

4. Wu P, Fang Y, Guan Z*, et al.* The Psychological Impact of the SARS Epidemic on Hospital Employees in China: Exposure, Risk Perception, and Altruistic Acceptance of Risk. *Can J Psychiatry* 2009; 54:302-311.https://doi.org/10.1177/070674370905400504

5. Li Y, Scherer N, Felix L*, et al.* Prevalence of depression, anxiety and post-traumatic stress disorder in health care workers during the COVID-19 pandemic: A systematic review and meta-analysis. *PLoS ONE* 2021; 16:e0246454.https://doi.org/10.1371/journal.pone.0246454

6. Moitra M, Rahman M, Collins PY*, et al.* Mental Health Consequences for Healthcare Workers During the COVID-19 Pandemic: A Scoping Review to Draw Lessons for LMICs. *Frontiers in Psychiatry* 2021; 12:22.https://www.frontiersin.org/article/10.3389/fpsyt.2021.602614

7. Vizheh M, Qorbani M, Arzaghi SM*, et al.* The mental health of healthcare workers in the COVID-19 pandemic: A systematic review. *Journal of Diabetes & Metabolic Disorders* 2020; 19:1967-1978.https://dx.doi.org/10.1007%2Fs40200-020-00643-9

8. Dunlop C, Howe A, Li D*, et al.* The coronavirus outbreak: the central role of primary care in emergency preparedness and response. *BJGP Open* 2020; 4:bjgpopen20X101041.https://pubmed.ncbi.nlm.nih.gov/31992543

9. Rawaf S, Allen LN, Stigler FL*, et al.* Lessons on the COVID-19 pandemic, for and by primary care professionals worldwide. *European Journal of General Practice* 2020; 26:129-133.https://doi.org/10.1080/13814788.2020.1820479

10. Sarti TD, Lazarini WS, Fontenelle LF*, et al.* Qual o papel da Aten+º+úo Prim+íria +á Sa+¦de diante da pandemia provocada pela COVID-19? *Epidemiologia e Serviços de Saúde* 2020; 29.https://doi.org/10.5123/s1679-49742020000200024

11. Bhaumik S, Moola S, Tyagi J*, et al.* Community health workers for pandemic response: a rapid evidence synthesis. *BMJ Global Health* 2020; 5:e002769.http://gh.bmj.com/content/5/6/e002769.abstract

For peer review only - http://bmjopen.bmj.com/site/about/guidelines.xhtml

12. Gray DM, II, Joseph JJ, Olayiwola JN. Strategies for Digital Care of Vulnerable Patients in a COVID-19 World–Keeping in Touch. *JAMA Health Forum* 2020; 1:e200734.https://doi.org/10.1001/jamahealthforum.2020.0734

13. First in, last out - The role of family doctors in the fight against novel coronavirus. 2020. World Organization of Family Doctors, WONCA. 7-10-2021.
Ref Type: Online Source

14. Mash B. Primary care management of the coronavirus (COVID-19). *S Afr Fam Pract (2004)* 2020; 62:e1-e4.https://pubmed.ncbi.nlm.nih.gov/32242438

15. Waters R. Community Workers Lend Human Connection To COVID-19 Response. *Health Aff (Millwood)* 2020; 39:1112-1117.https://doi.org/10.1377/hlthaff.2020.00836

16. Donnelly C, Ashcroft R, Bobbette N*, et al.* Interprofessional primary care during COVID-19: a survey of the provider perspective. *BMC Family Practice* 2021; 22.https://doi.org/10.1186%2Fs12875-020-01366-9

17. Ashcroft R, Sur D, Greenblatt A*, et al.* The Impact of the COVID-19 Pandemic on Social Workers at the Frontline: A Survey of Canadian Social Workers. *The British Journal of Social Work* 2021; 52:1724-1746.https://doi.org/10.1093/bjsw/bcab158

18. Strengthening the frontline: How primary health care helpshealth systems adapt during the COVID-19 pandemic. 10-2-2021. OECD Policy Responses to Coronavirus (COVID-19). 12-5-2021.
Ref Type: Report

19. National Center for Immunization and Respiratory Diseases (NCIRD) Division of Viral Diseases. Engaging Community Health Workers to Support Home-based Care for People with COVID-19 in Low-Resource Non-U.S. Settings. 7-4-2021. United States, Center for Disease Control and Prevention. 12-5-2021.
Ref Type: Online Source

20. De Kock JH, Latham HA, Leslie SJ*, et al.* A rapid review of the impact of COVID-19 on the mental health of healthcare workers: implications for supporting psychological well-being. *BMC Public Health* 2021; 21:104.https://doi.org/10.1186/s12889-020-10070-3

21. Tremblay S, Castiglione S, Audet LA*, et al.* Conducting Qualitative Research to Respond to COVID-19 Challenges: Reflections for the Present and Beyond. *International Journal of Qualitative Methods* 2021; 20:16094069211009679.https://doi.org/10.1177/16094069211009679

22. Zace D, Hoxhaj I, Orfino A*, et al.* Interventions to address mental health issues in healthcare workers during infectious disease outbreaks: A systematic review. *J Psychiatr Res* 2021; 136:319-333.https://www.sciencedirect.com/science/article/pii/S0022395621000868

23. Torjesen I. Covid-19 will become endemic but with decreased potency over time, scientists believe. *BMJ* 2021; 372:n494.http://www.bmj.com/content/372/bmj.n494.abstract

For peer review only - http://bmjopen.bmj.com/site/about/guidelines.xhtml

884 24. Phillips N. The coronavirus is here to stay — here's what that means. *Nature* 2021; 590:382-384.https://doi.org/10.1038/d41586-021-00396-2

886 25. Gunnell D, Appleby L, Arensman E*, et al.* Suicide risk and prevention during the COVID-19 pandemic. *The Lancet Psychiatry* 2020.https://doi.org/10.1016/S2215-0366(20)30171-1

888 26. Population Reference Bureau. 2021 World Population Data Sheet. 2021. Washington DC, Population Reference Bureau. 28-9-2021.
890 Ref Type: Online Source

891 27. National Information Technology Board. COVID-19 Dashboard. 27-4-2020. Islamabad, Government of Pakistan. 27-4-2020.
893 Ref Type: Online Source

894 28. Hassan A, Mahmood K, Bukhsh HA. Healthcare System Of Pakistan. *International Journal of Advanced Research and Publication* 2017; 1:170-173.https://www.ijarp.org/published-research-papers/oct2017/Healthcare-System-Of-Pakistan.pdf

897 29. Government of Punjab. National Programme for Family Planning & Primary Health Care, Pakistan. 2011. National Programme for Family Planning & Primary Health Care. 4-5-2015.
899 Ref Type: Online Source

900 30. Waris A, Atta UK, Ali M*, et al.* COVID-19 outbreak: current scenario of Pakistan. *New Microbes and New Infections* 2020; 35:100681.http://www.sciencedirect.com/science/article/pii/S2052297520300330

903 31. Khalid A, Ali S. COVID-19 and its Challenges for the Healthcare System in Pakistan. *Asian Bioethics Review* 2020; 12:551-564.https://dx.doi.org/10.1007%2Fs41649-020-00139-x

905 32. Fullman N, Yearwood J, Abay SM*, et al.* Measuring performance on the Healthcare Access and Quality Index for 195 countries and territories and selected subnational locations: a systematic analysis from the Global Burden of Disease Study 2016. *Lancet* 2018; 391:2236-2271.https://doi.org/10.1016/S0140-6736(18)30994-2

909 33. Abdullah MA, Mukhtar F, Wazir S*, et al.* The Health Workforce Crisis in Pakistan: A Critical Review and the Way Forward. *World Health & Population* 2014; 15:4-12.https://www.longwoods.com/product/24031

912 34. Asim SS, Ghani S, Ahmed M*, et al.* Assessing Mental Health of Women Living in Karachi During the Covid-19 Pandemic. *Frontiers in Global Women's Health* 2021; 1:594970.https://doi.org/10.3389/fgwh.2020.594970

915 35. Sandesh R, Shahid W, Dev K*, et al.* Impact of COVID-19 on the Mental Health of Healthcare Professionals in Pakistan. *Cureus* 2020; 12:e8974.https://pubmed.ncbi.nlm.nih.gov/32775056

917 36. Arshad MS, Hussain I, Nafees M*, et al.* Assessing the Impact of COVID-19 on the Mental Health of Healthcare Workers in Three Metropolitan Cities of Pakistan. *Psychol Res Behav Manag* 2020; 13:1047-1055.https://pubmed.ncbi.nlm.nih.gov/33244279

For peer review only - http://bmjopen.bmj.com/site/about/guidelines.xhtml

37. Perry H, Zulliger R, Scott K, Javadi D, Gergen J, Shelley K et al. Case Studies of Large-Scale Community Health Worker Programs: Examples from Afghanistan, Bangladesh, Brazil, Ethiopia, Niger, India, Indonesia, Iran, Nepal, Pakistan, Rwanda, Zambia, and Zimbabwe. 2017. Baltimore, United States, Maternal and Child Health Integrated Program. 1-9-2022.
Ref Type: Report

38. Oxford Policy Management. Lady Health Worker Programme: External Evaluation of the National Programme for Family Planning and Primary Health Care. 2009. United Kingdom, Oxford Policy Management.
Ref Type: Report

39. Bechange S, Schmidt E, Ruddock A*, et al.* Understanding the role of lady health workers in improving access to eye health services in rural Pakistan - findings from a qualitative study. *Arch Public Health* 2021; 79.https://doi.org/10.1186/s13690-021-00541-3

40. Creswell JW, Poth CN. Qualitative Inquiry and Research Design: Choosing Among Five Approaches. Fourth Edition ed. SAGE Publishing; 2017.

41. Yin RK. Case Study Research and Applications: Design and Methods. Sixth Edition ed. SAGE Publishing; 2017.

42. National Information Technology Board. COVID-19 Dashboard. 30-8-2022. Islamabad, Government of Pakistan. 30-8-2022.
Ref Type: Online Source

43. Fusch PI, Ness LR. Are We There Yet? Data Satur et? Data Saturation in Qualitative Research. *The Qualitative Report* 2015; 20:1408-1416.https://nsuworks.nova.edu/cgi/viewcontent.cgi?article=2281&context=tqr

44. NVivo qualitative data analysis software [QSR International Pty Ltd.; 2015.

45. Hsieh HF, Shannon SE. Three Approaches to Qualitative Content Analysis. *Qual Health Res* 2005; 15:1277-1288.https://doi.org/10.1177/1049732305276687

46. Tong A, Sainsbury P, Craig J. Consolidated criteria for reporting qualitative research (COREQ): a 32-item checklist for interviews and focus groups. *Int J Qual Health Care* 2007; 19:349-357.https://doi.org/10.1093/intqhc/mzm042

47. Su Z, McDonnell D, Wen J*, et al.* Mental health consequences of COVID-19 media coverage: the need for effective crisis communication practices. *Globalization and Health* 2021; 17:4.https://doi.org/10.1186/s12992-020-00654-4

48. World Health Organization. Health workforce policy and management in the context of the COVID-19 pandemic response: interim guidance, 3 December 2020. 3-12-2020. Geneva, World Health Organization. 10-10-2021.
Ref Type: Report

For peer review only - http://bmjopen.bmj.com/site/about/guidelines.xhtml

49. Amin F, Sharif S, Saeed R, *et al.* COVID-19 pandemic- knowledge, perception, anxiety and depression among frontline doctors of Pakistan. *BMC Psychiatry* 2020; 20:459.https://doi.org/10.1186/s12888-020-02864-x

50. Hoernke K, Djellouli N, Andrews L, *et al.* Frontline healthcare workers' experiences with personal protective equipment during the COVID-19 pandemic in the UK: a rapid qualitative appraisal. *BMJ Open* 2021; 11:e046199.http://bmjopen.bmj.com/content/11/1/e046199.abstract

51. Martins RS, Bhatti OA, Mian AI. Violence Against Health Care Workers in Pakistan During the COVID-19 Pandemic. *JAMA Health Forum* 2020; 1:e201263.https://doi.org/10.1001/jamahealthforum.2020.1263

52. Bhatti OA, Rauf H, Aziz N, *et al.* Violence against Healthcare Workers during the COVID-19 Pandemic: A Review of Incidents from a Lower-Middle-Income Country. *Ann Glob Health* 2021; 87:41.https://pubmed.ncbi.nlm.nih.gov/33977084

53. Shaikh S, Baig LA, Hashmi I, *et al.* The magnitude and determinants of violence against healthcare workers in Pakistan. *BMJ Global Health* 2020; 5:e002112.http://gh.bmj.com/content/5/4/e002112.abstract

54. Al-Mansour K, Alfuzan A, Alsarheed D, *et al.* Work-Related Challenges among Primary Health Centers Workers during COVID-19 in Saudi Arabia. *Int J Environ Res Public Health* 2021; 18:1898.https://pubmed.ncbi.nlm.nih.gov/33669440

55. Labrague LJ. Psychological resilience, coping behaviours and social support among health care workers during the COVID-19 pandemic: A systematic review of quantitative studies. *J Nurs Manag* 2021; n/a.https://doi.org/10.1111/jonm.13336

56. Ministry of National Health Services Regulation and Coordination (MoNHSRC). We CARE. 2020. Islamabad, Government of Pakistan. 18-9-2021.
Ref Type: Online Source

57. Mistry SK, Harris-Roxas B, Yadav UN, *et al.* Community Health Workers Can Provide Psychosocial Support to the People During COVID-19 and Beyond in Low- and Middle- Income Countries. *Frontiers in Public Health* 2021; 9:800.https://www.frontiersin.org/article/10.3389/fpubh.2021.666753

58. Shaukat N, Ali DM, Razzak J. Physical and mental health impacts of COVID-19 on healthcare workers: a scoping review. *Int J Emerg Med* 2020; 13:40.https://pubmed.ncbi.nlm.nih.gov/32689925

59. Donmoyer R. Generalizability and the Single-Case Study. In: Gomm R, Hammersley M, Foster P, editors. Case Study Method. SAGE publications Ltd.; 2009.

For peer review only - http://bmjopen.bmj.com/site/about/guidelines.xhtml

COVID-19: psychosocial and mental health needs of health workers during the outbreak management in Pakistan

IN-DEPTH INTERVIEW GUIDE FOR HEALTH MANAGERS AND SERVICE PROVIDERS

SECTION 1: PARTICIPANT CONTACT SHEET			
**S.no**	**Questions**	**Responses**	**Skip**
Please complete before the interview.			
101	Name of province	Sindh 1<br>Punjab 2	
102	Name of district		
103	Name of TALUKA / Tehsil		
104	Name of Union Council		
105	Type of health facility	Basic Health Unit (BHU) 1<br>Rural Health Centre (RHC) 2<br>Taluka Head Quarter (THQ) 3<br>District Head Quarter (DHQ) 4<br>Tertiary care hospital 5<br>Specialised Isolation Centre 6	
106	Name of health facility / Hospital		
107	Date of interview	______ / _______ / _____________<br>dd mm yyyy	
**SECTION 2: PARTICIPANTS PROFILE**			
201	Sex	Male 1<br>Female 2	
202	Marital status	Never married 1<br>Currently married 2<br>Divorced 3<br>Separated 4<br>Widowed 5	
203	Age (in years)	Years [ ][ ]	
204	Number of living children	Children [ ][ ]	
205	Do you live in a nuclear family or joint family?	Nuclear 1<br>Joint 2	
206	Highest level of education completed?	Matriculation 1<br>Intermediate 2<br>Bachelors 3<br>Masters / M.Phil. 4<br>PhD 5	
207	Professional qualification	Lady Health Visitor 1<br>Lady Health Worker 2	

Version 1.0 Last updated 29th June 2020

For peer review only - http://bmjopen.bmj.com/site/about/guidelines.xhtml

		Midwife 3<br>Nurse 4<br>M.B.B.S 5	
208	What is the current position you hold in the organization?	Lady health supervisors (LHSs) 1<br>Managers from secondary level health facility (DHQ/THQ) 2<br>Medical superintendent of tertiary care teaching hospital 3<br>Specialist from Field isolation center, Karachi 4<br>Others specify______________________ 5	
209	How long you have been working in this health facility / catchment area?	Years [ ][ ]	
210	What is the total years of your professional experience?	Years [ ][ ]	

For peer review only

For peer review only - http://bmjopen.bmj.com/site/about/guidelines.xhtml

No.	Questions	Responses	Probe
**1**	**THEME : Understanding psychological impact of covid-19 on healthcare workers**		
	**First, I would like to ask you some questions to understand the psychological impact of covid-19 on healthcare workers**<br>**The psychological adverse outcomes may include stress, anxiety, depression, burnout, post-traumatic stress disorder, fear-triggered panic attacks, psychosomatic symptoms, psychological violence, fatigue, stigma etc.**		
1.1	How healthcare workers feel about working under tremendous pressure with COVID -19 situation?		Probes:<br>• Can you use example to explain the situation?
1.2	In your opinion, how has COVID-19 affected mental health of healthcare workers?		Probes:<br>• How healthcare workers are dealing with anxiety and fear related to managing COVID-19 inpatients?<br>• Is it affecting healthcare workers both negatively and positively?
1.3	Can you specify the reasons for adverse psychological outcomes among healthcare workers?		Probes:<br>• Reasons related to procedural changes (increased workload, long working hours)
1.4	How negative emotions among healthcare workers will affect the overall functioning of organisations/ institutions?		Probes:<br>• Do you think negative emotions among health worker may lead to poor health worker attention, understanding and decision-making ability
1.5	Do you think health workers have capacity to cope with psychological risk?		Probes:<br>• If yes, what are the coping mechanisms of healthcare workers?<br>• If no, why do you think healthcare workers lack capacity to cope with psychological risk?
1.6	Do you think psychological needs of population has changed in recent times?		Probes:<br>• How health system is responding to those psychological needs?<br>• Are health workers prepared to address changing psychological needs of patients with covid-19?
**2**	**THEME: Identifying psychological needs of health workforce involved in covid-19 crisis**		
	**Now I would like to ask some questions to understand the psychological needs of health workforce involved in covid-19 crisis. The psychological needs of healthcare workers may include enabling working environment, safe and secured hospital environment, adequate staffing, support from mentors and floor managers, support from mental health counsellors, etc.**		
2.1	What are the psychological needs of health workforce involved in covid-19 crisis		Probes:<br>• How did you get to know about their psychological needs?

For peer review only - http://bmjopen.bmj.com/site/about/guidelines.xhtml

2.2	Do you think healthcare workers are able to talk about their psychological needs and concerns?		Probes:<br>• If yes, please share examples of some instances where healthcare workers have shared their psychological needs<br>• If no, why healthcare workers are not able to talk about their concerns?
2.3	As a ______, how do you see your role in identifying the psychological needs of the health workforce		
**3**	**THEME: Support mechanisms available to address mental health needs of healthcare workers**		
	I would like to know the support mechanisms/ processes available to address mental health needs of healthcare workers. These support mechanisms may include providing mental health support services, delivering counselling sessions, supporting workers through mental health helpline, etc.		
3.1	What are the mechanisms available to support the mental health needs of healthcare workers?		Probes:<br>• What interventions are currently used to support the mental health needs of healthcare workers?<br>•
3.2	Do you think healthcare workers are able to receive adequate mental health support with these processes?		Probes:<br>• Are they effective? Are these interventions appreciated by current health workforce?
3.3	Do you think your institution/health facility is prepared to manage mental health needs of the healthcare workers?		Probes:<br>• If yes, please share a recent experience whereby mental health needs of a health worker were adequately addressed.<br>• Do you think health system is currently prepared to address the mental health needs of health care workers<br>○ If yes, why? If no, why?
3.4	Have you received any formal training/ session on supporting mental health needs of the healthcare workers?		Probes:<br>• How many times the training was conducted and for whom?<br>• Can you tell me the objective of each training?<br>• What aspects were covered in the training?<br>• Who conducted those trainings?<br>• What was the duration of each training?
3.5	As a ______, how do you see your role in addressing the psychological needs of the health workforce		Probes:<br>• How do you provide the support to your staff?<br>• Do you feel confident while addressing the mental health needs of your staff?

For peer review only - http://bmjopen.bmj.com/site/about/guidelines.xhtml

For peer review only

			• How do you feel about your capacity in terms of addressing psychological needs of your staff<br>• How effective it is?
4	**Theme: Understanding challenges in addressing mental health needs of healthcare workers**		
	Now I would like to understand the challenges you face while addressing mental health needs of health care workers like lack of adequate trainings, tools and resources		
4.1	What are some of the barriers you face in addressing mental health needs of healthcare workers?		Probes:<br>• Do you think lack of trainings/resources as one of the barriers to addressing mental health needs of healthcare workers?<br>• How health system can address these barriers?
4.2	In your opinion, how these barriers could be handled efficiently, to ensure adequate mental health support for healthcare workers?		
5	**Theme: Suggestions and recommendations to address psychological needs of health care workers**		
	Here I would like to understand how institutions could provide mental health support to healthcare workers. Specifically, we would like to understand the interventions that the health system can offer to alleviate psychological burden of healthcare workers.		
5.1	What kind of relief healthcare workers expect to manage with these pressures?		
5.2	Do you have suggestions on how institutions could provide mental health support services for coping with stress related to this pandemic		Probes:<br>• How health system is prepared to address psychological needs of healthcare workers?<br>• How health system can ensure implementation of process and tolls to support mental health needs of healthcare workers?<br>• How health system will sustain the mental health support activities for its healthcare workers?
5.3	Do you have suggestions on how provincial/federal government could provide mental health support services for coping with stress related to this pandemic		

For peer review only - http://bmjopen.bmj.com/site/about/guidelines.xhtml

COREQ (COnsolidated criteria for REporting Qualitative research) Checklist

A checklist of items that should be included in reports of qualitative research. You must report the page number in your manuscript where you consider each of the items listed in this checklist. If you have not included this information, either revise your manuscript accordingly before submitting or note N/A.

Topic	Item No.	Guide Questions/Description	Reported on Page No.
**Domain 1: Research team and reflexivity**			
Personal characteristics			
Interviewer/facilitator	1	Which author/s conducted the interview or focus group?	7
Credentials	2	What were the researcher's credentials? E.g. PhD, MD	7
Occupation	3	What was their occupation at the time of the study?	7
Gender	4	Was the researcher male or female?	7
Experience and training	5	What experience or training did the researcher have?	7
Relationship with participants			
Relationship established	6	Was a relationship established prior to study commencement?	6
Participant knowledge of the interviewer	7	What did the participants know about the researcher? e.g. personal goals, reasons for doing the research	6
Interviewer characteristics	8	What characteristics were reported about the inter viewer/facilitator? e.g. Bias, assumptions, reasons and interests in the research topic	N/A
**Domain 2: Study design**			
Theoretical framework			
Methodological orientation and Theory	9	What methodological orientation was stated to underpin the study? e.g. grounded theory, discourse analysis, ethnography, phenomenology, content analysis	7
Participant selection			
Sampling	10	How were participants selected? e.g. purposive, convenience, consecutive, snowball	6
Method of approach	11	How were participants approached? e.g. face-to-face, telephone, mail, email	6
Sample size	12	How many participants were in the study?	6
Non-participation	13	How many people refused to participate or dropped out? Reasons?	7
Setting			
Setting of data collection	14	Where was the data collected? e.g. home, clinic, workplace	6
Presence of nonparticipants	15	Was anyone else present besides the participants and researchers?	7
Description of sample	16	What are the important characteristics of the sample? e.g. demographic data, date	8
Data collection			
Interview guide	17	Were questions, prompts, guides provided by the authors? Was it pilot tested?	6
Repeat interviews	18	Were repeat inter views carried out? If yes, how many?	7
Audio/visual recording	19	Did the research use audio or visual recording to collect the data?	7
Field notes	20	Were field notes made during and/or after the interview or focus group?	7
Duration	21	What was the duration of the inter views or focus group?	7
Data saturation	22	Was data saturation discussed?	7

For peer review only

Transcripts returned	23	Were transcripts returned to participants for comment and/or correction?	6
**Domain 3: analysis and findings**			
Data analysis			
Number of data coders	24	How many data coders coded the data?	7
Description of the coding tree	25	Did authors provide a description of the coding tree?	7
Derivation of themes	26	Were themes identified in advance or derived from the data?	7
Software	27	What software, if applicable, was used to manage the data?	7
Participant checking	28	Did participants provide feedback on the findings?	7
Reporting			
Quotations presented	29	Were participant quotations presented to illustrate the themes/findings? Was each quotation identified? e.g. participant number	7
Data and findings consistent	30	Was there consistency between the data presented and the findings?	NA
Clarity of major themes	31	Were major themes clearly presented in the findings?	8
Clarity of minor themes	32	Is there a description of diverse cases or discussion of minor themes?	8

Developed from: Tong A, Sainsbury P, Craig J. Consolidated criteria for reporting qualitative research (COREQ): a 32-item checklist for interviews and focus groups. International Journal for Quality in Health Care. 2007. Volume 19, Number 6: pp. 349 – 357

Once you have completed this checklist, please save a copy and upload it as part of your submission. DO NOT include this checklist as part of the main manuscript document. It must be uploaded as a separate file.

For peer review only
